# Binge alcohol drinking alters the differential control of cholinergic interneurons over nucleus accumbens D1 and D2 medium spiny neurons

**DOI:** 10.3389/fncel.2022.1010121

**Published:** 2022-12-15

**Authors:** Jenya Kolpakova, Vincent van der Vinne, Pablo Gimenez-Gomez, Timmy Le, Gilles E. Martin

**Affiliations:** ^1^Department of Neurobiology, Brudnick Neuropsychiatric Research Institute, University of Massachusetts Chan Medical School, Worcester, MA, United States; ^2^Graduate Program in Neuroscience, Morningside Graduate School of Biomedical Sciences, University of Massachusetts Chan Medical School, Worcester, MA, United States; ^3^Department of Biology, Williams College, Williamstown, MA, United States

**Keywords:** nucleus accumbens, cholinergic interneuron, optogenetic, glutamatergic synaptic transmission, dopamine

## Abstract

Animals studies support the notion that striatal cholinergic interneurons (ChIs) play a central role in basal ganglia function by regulating associative learning, reward processing, and motor control. In the nucleus accumbens (NAc), a brain region that mediates rewarding properties of substance abuse, acetylcholine regulates glutamatergic, dopaminergic, and GABAergic neurotransmission in naïve mice. However, it is unclear how ChIs orchestrate the control of these neurotransmitters/modulators to determine the synaptic excitability of medium spiny neurons (MSNs), the only projecting neurons that translate accumbens electrical activity into behavior. Also unknown is the impact of binge alcohol drinking on the regulation of dopamine D1- and D2 receptor-expressing MSNs (D1- and D2-MSNs, respectively) by ChIs. To investigate this question, we optogenetically stimulated ChIs while recording evoked and spontaneous excitatory postsynaptic currents (sEPSCs) in nucleus accumbens core D1- and D2-MSN of ChAT.ChR2.eYFPxDrd1.tdtomato mice. In alcohol-naïve mice, we found that stimulating NAc ChIs decreased sEPSCs frequency in both D1- and D2-MSNs, presumably through a presynaptic mechanism. Interestingly, ChI stimulation decreased MSN synaptic excitability through different mechanisms in D1- vs. D2-MSNs. While decrease of ChI-mediated sEPSCs frequency in D1-MSNs was mediated by dopamine, the same effect in D2-MSNs resulted from a direct control of glutamate release by ChIs. Interestingly, after 2 weeks of binge alcohol drinking, optogenetic stimulation of ChIs enhanced glutamate release in D1-MSNs, while its effect on D2-MSNs remained unchanged. Taken together, these data suggest that cholinergic interneurons could be a key target for regulation of NAc circuitry and for alcohol consumption.

## Introduction

Addiction is a disorder of the reward system (Koob et al., 1994; Koob and Volkow, 2016) where drugs of abuse distort the response to natural reinforcers leading to continued drug use, which, in turn, impairs brain function by interfering with the capacity to exert self-control over drug-taking behaviors such as binge drinking (Koob et al., 1994; Koob and Volkow, 2016). Binge alcohol drinking is the main mode of alcohol consumption in late adolescents and young adults and often serves as a gateway to alcohol dependence later in life (Crabbe et al., 2011). One of the main brain areas controlling drug taking behaviors is the nucleus accumbens (NAc), a forebrain region that encodes association between temporally unpredictable stimuli and the appropriate action to maximize reward or avoid punishment (Nicola, 2007). MSNs expressing dopamine-1 and 2 receptors (D1- and D2-MSNs) are the sole output neurons of the NAc. In a recent study, Strong et al. (2020) showed that optogenetics-mediated inhibition of D1- and D2-MSNs decreased and increased alcohol consumption, respectively, in both males and females (Strong et al., 2020), and optogenetic manipulation of their excitability has been causally linked to reward-seeking behaviors (Ma et al., 2014; Soares-Cunha et al., 2020). The role traditionally attributed to MSNs is that of integrators that receive a range of different inputs (glutamate, dopamine, acetylcholine, and GABA) from across the brain and determine the optimal behavioral response (Humphries and Prescott, 2010; Francis et al., 2019; Soares-Cunha et al., 2020). In recent years, this view has been challenged by the observations that the integration of different inputs is mainly performed by a different cell population in the NAc: Cholinergic interneurons (ChIs) (Lim et al., 2014; Abudukeyoumu et al., 2019).

Cholinergic interneurons (ChIs) make up only 1–2% of all neurons in the striatum (Dopico et al., 1998), but play an outsize role in regulating NAc GABAergic (Melendez-Zaidi et al., 2019), glutamatergic (Higley et al., 2011; Assous, 2021) and dopaminergic synaptic transmission (Threlfell et al., 2012; Collins et al., 2016) through their extensive projections (Lim et al., 2014). NAc ChIs generate unique bidirectional outcome responses during reward-based learning, signaling both positive (reward) and negative (reward omission) outcomes (Atallah et al., 2014). Cholinergic receptor signaling has been shown to alter alcohol and other drugs’ consumption (Rahman and Prendergast, 2012; Hendrickson et al., 2013; Scofield et al., 2016). Currently, both the role played by ChIs in orchestrating dopamine (DA) and glutamatergic synaptic transmission to regulate D1- and D2-MSNs synaptic excitability, as well as how alcohol exposure modulates this connection remain to be elucidated. Here we combine *in vitro* patch clamp, fast scan cyclic voltammetry (FSCV), optogenetics, and behavioral recordings to answer these questions.

In alcohol-naïve mice, we demonstrate that optogenetic stimulation of ChIs decreases the frequency of spontaneous excitatory post-synaptic currents (sEPSCs), presumably through a presynaptic mechanism, in both nucleus accumbens core D1- and D2-MSNs. In D1-MSNs, inhibition of glutamatergic synaptic transmission by ChIs is mediated by dopaminergic and cholinergic (nAChR and mAChR) receptors. Although ChIs induced a similar effect on sEPSPCs in D2-MSNs, this effect did not require DA. Instead, glutamatergic inhibition likely resulted from ChIs synapsing directly on glutamatergic terminals. Importantly, binge alcohol drinking differentially altered ChIs control of glutamatergic synaptic transmission in D1- and D2-MSNs. While the ChI-mediated decrease of sEPSCs frequency in D2-MSNs was unaffected, the ChI-induced inhibition of glutamatergic transmission in D1-MSNs seen in naïve mice was reversed and optogenetic stimulation became potentiating following alcohol exposure. Our findings elucidate mechanisms by which ChIs differentially control synaptic excitability of D1- and D2-MSNs in naïve and alcohol conditions, and their influence on binge alcohol drinking.

## Materials and methods

### Animals

All experiments were performed using heterozygous male ChAT.ChR2.eYFPxDrd1.tdtomato mice of C57Bl/6J background. All mice were handled according to the American Association for the Accreditation of Laboratory Animal Care guideline. The protocol was approved by the Institutional Animal Care and Use Committee of University of Massachusetts Medical School. Mice were maintained at constant temperature (22 ± 1°C) and humidity with a 12 h:12 h light–dark cycle. Water and food were provided *ad libitum*.

### Immunostaining

Mice were euthanized using pentobarbital (120 mg/kg, i.p) followed by transcardiac perfusion with 0.1 M phosphate buffer followed by 4% p-formaldehyde (PFA) in 0.1 M phosphate buffer (pH 7.4). Brains were removed, post-fixed in 4% PFA overnight and placed in 30% sucrose solution for 48 h. Coronal series sections (20 μm) were sliced on a freezing microtome (Leica SM2000R, Leica Microsystems GmbH, Buffalo Grove, IL, USA) and stored in a cryoprotective solution. Double-label immunofluorescence was performed on free-floating sections and incubated overnight at 4°C with the following primary antibodies: Goat anti-chicken GFP (ab13970, Abcam, Cambridge, United Kingdom) and Goat anti-rabbit RFP (ab185921, Abcam, Cambridge, United Kingdom). Secondary antibodies used were Goat anti-chicken Alexa Fluor 488 (A-11039, Thermo Fisher, Waltham, MA, USA) and Goat anti-rabbit Alexa Fluor 594 (A11007, Thermo Fisher, Waltham, MA, USA). Sections were counter stained with 4′, 6-diamidino-2-phenylindole (DAPI) (Sigma, D9564). After this incubation, sectioned were washed, mounted and coverslipped. Controls performed in parallel without primary antibodies showed very low levels of non-specific staining. Image acquisition was performed with a laser-scanning confocal imaging system (Zeiss LSM710) and image analysis was performed with the ZEN 2009 software (Zeiss, Oberkochen, Germany).

### Slice preparation

Slices were prepared according to method previously described (Kolpakova et al., 2021). Briefly, we prepared coronal slices from fresh brain tissue of 8–9 weeks old mice. Following intracardiac perfusion with an ice-cold *N*-methyl-D-glucamine-based solution (see below), we rapidly removed and transferred the brain in a cold (∼0°C) oxygenated (95% O_2_ and 5% CO_2_) cutting solution of the following composition (in mM): 92 *N*-methyl-D-glucamine (NMDG), 2.5 KCl, 1.25 NaH2PO4.H20, 30 NaHCO3, 20 HEPES, 25 Glucose, 2 Thiourea, 5 Na+-ascorbate, 3 Na+-pyruvate, 0.5 CaCl2.2H2O, 10 MgSO4.7H2O, pH 7.37. Slices were cut 200 μm thick with a Vibroslicer (VT1200, Leica MicroInstrutments; Germany). Slices were immediately transferred to an incubation chamber and left to recuperate in the NMDG-based solution for 20–30 min at 32°C before being moved into a chamber containing an oxygenated artificial cerebrospinal fluid (ACSF; in mM): 126 NaCl, 2.5 KCl, 1.25 NaH2PO4.H2O, 1 MgCl2.H20, 2 CaCl2H2O, 26 NaHCO3, 10 D-Glucose, at room temperature. Slices were left in this chamber for at least 1 h before being placed in a recording chamber and perfused with ACSF at a constant rate of 2–3 ml/min at room temperature (∼21°C).

### Fast-scan cyclic voltammetry

Striatal slices were prepared as described for *ex vivo* slice biotinylation and recovered at 31°C for a minimum of 1 h prior to recording in oxygenated ASCF supplemented with 500 μM Na-Ascorbate. Glass pipettes containing a 7 μm carbon-fiber microelectrode were prepared and preconditioned in ASCF by applying triangular voltage ramps (–0.4 to +1.2 and back to –0.4 V at 400 V/s), delivered at 60 Hz for 1 h. Recordings were performed at 10 Hz. Electrodes were calibrated to a 1 μM DA standard prior to recording. Electrodes were positioned in DS and DA transients were electrically evoked with a 250 μA rectangular pulse every 2 min, using a concentric bipolar electrode placed ∼100 μm from the carbon fiber electrode. Data were collected with a 3-electrode headstage, using an EPC10 amplifier (Heka, Harvard Bioscience Holliston, MA, USA) after low-pass filter at 10 kHz and digitized at 100 kHz, using Patchmaster software (Heka, Harvard Bioscience Holliston, MA, USA). A stable baseline was achieved after evoking six consecutive DA transients, after which experimental data were collected. Each biological replicate is the average of three evoked DA transients/slice, and a minimum of three independent mice were used to gather data from the indicated number of slices in each experiment. Data were analyzed in Igor Pro, using the Wavemetrics FSCV plugin (gift of Veronica Alvarez, NIAAA). Peak amplitudes were measured for each individual DA transient, and tau was calculated as 1/e according to the equation: y = y_0_ + A^[(x–x^_0_^)/tau]^.

### Electrophysiology

Whole-cell patch clamp recordings of spontaneous excitatory post-synaptic currents (sEPSCs), and electrically evoked excitatory post-synaptic potentials (EPSPs) in MSNs in the NAc were performed in the presence of 15 μM GABA receptor antagonist Bicuculline. NAc MSNs were visualized in infrared differential interference contrast videomicroscopy using a fully motorized microscope mounted with 10x and 60x objective (Olympus Microscopy, Shinjuku City, Tokyo, Japan), and tdTomato-D1R MSNs were identified by fluorescence microscopy. Recordings were performed according to the method described previously (Ji et al., 2017). Briefly, borosilicate glass electrodes (1.5 mm OD, 4–6 MΩ resistance) were filled with an internal solution containing (mM): 120 K-methanesulfonate; 20 KCl; 10 HEPES; 2 ATP, 1 GTP, and 12 phosphocreatine. Following seal rupture, series resistance was 18.3 ± 1.1 MΩ in a randomly selected sample of 23 MSNs, fully compensated and periodically monitored throughout recording sessions. Recordings with changes of series resistance larger than 20% were rejected, as were MSNs with a resting membrane potential more positive than –80 mV. Voltage and current traces in whole-cell patch-clamp were acquired with an EPC10 amplifier (HEKA Elektronik, Lambrecht, Germany). Sampling was performed at 10 kHz and digitally filtered voltage and current traces were acquired with PatchMaster 2.15 (HEKA Elektronik, Lambrecht, Germany) at 2 kHz. All traces were subsequently analyzed off-line with FitMaster 2.15 (HEKA Electronik; Germany). We analyzed sEPSCs amplitude and frequency with Clampfit (pClamp 11 Software suite, Molecular Devices, CA). We monitored series resistance by comparing EPSPs decay time before and after induction using Clampfit event template analysis. Spontaneous EPSCs, measured at MSNs resting membrane potentials (i.e., around −85 mV), were acquired for 4–6 min before (Pre) and 4 min after (Post) optogenetic stimulation using gap-free recording at MSN resting membrane potential. Then, cholinergic interneurons were stimulated optogenetically by flashing a train of five 1 ms-long pulses at 20 Hz every 20 s for 2 min at 470 nm through the light path of a microscope 60x objective using independent high-powered LEDs (pE-100 470 CooLED, NY, USA) under the control of the acquisition software (PatchMaster, HEKA, Germany). Antagonists were added to the recording bath at the final slice concentration of (in μM): 1 Atropine, 5 Mecamylamine, 5 SCH-23390, 1 Sulpiride. When recording electrically evoked EPSPs, we positioned a bipolar concentric stimulating electrode (FHC, Bowdoin, ME, USA) in the vicinity (i.e., ∼50–100 μm) of MSNs and delivered constant current pulses (100 μs and 10–20 μA). All electrophysiological experiments required between 4 and 5 mice (9–14 cells) per experimental group.

#### Drinking in the dark paradigm

Two days following brain viral injection, individually housed mice were allowed to adapt to a reversed light-dark cycle (12 h cycle, OFF at 7 a.m., ON at 7 p.m.) for 1 week. Mice were given water bottles with sipper tubes before the experiment to allow habituation and reduce the novelty effect once the ethanol bottle, containing a similar sipper tube, was presented. The total habituation time for the reverse light-dark cycle was 2 weeks before the experiment began. Ethanol exposure started 2 h into the dark phase and lasted for 2 h (Rhodes et al., 2005; Hendrickson et al., 2009). At the start of the experiment, each water bottle was removed and replaced with a pre-weighed 50-mL conical tube containing 20% ethanol with a rubber stopper and double-ball bearing sipper tube. Mice were allowed to drink for 2 h, and then the ethanol bottles were removed, weighed and the water bottles were returned. Ethanol consumed was measured as grams ethanol divided by mouse body weight in kilograms. This protocol was repeated 5 days a week with 2 days off (water only) after each 5-day span. Drip controls were used to account for evaporation and dripping, and experimental bottle weights were corrected using these control values. On average, mice drink around 3 g/kg on the start of the DID protocol (Supplementary Figure 6). All electrophysiological recordings were performed 24 h after the last drinking bout during the 4th or 5th post-surgery weeks. Supplementary Figure 6 shows average alcohol consumption.

#### Optogenetic stimulation in freely moving mice

Four-week old mice were anesthetized and implanted with an optic fiber cannula (Doric Lenses, Quebec, Canada) located above the NAc (AP + 1.5, ML ± 1.5, DV –4.0 mm from Bregma). We checked optic fiber placement at the end of experiments (Figure 7B). Mice were allowed to recover for 2–3 weeks in the reverse reversed light-dark cycle (12 h cycle, OFF at 7 a.m., ON at 7 p.m.). Mice were handled every day for 2 weeks prior to initiation of the experiment. Twenty-four hours prior to the first alcohol exposure mice were connected to the fiber optic cable for habituation purposes, however, during consecutive stimulation days mice were connected only for an hour prior to beginning of the stimulation each day. Mice received the same pattern of optogenetic stimulation as during the electrophysiologic recordings: a burst of five 2-ms long 470 nm light pulses at 20 Hz every 20 s. Initial stimulations started 2 min prior to first alcohol exposure and then continued for the duration of alcohol drinking session of 1 h. We limited alcohol consumption concurrent with stimulation to 1 h, as the first hour of alcohol exposure contained majority of alcohol consumption (Kolpakova et al., 2021). Mice were stimulated every day for four consecutive days. We quantified drinking behavior by measuring the number and timing of licks. The comparison was made between the optogenetically stimulated ChAT-ChR2-eYFP mice, non-stimulated ChAT-ChR2-eYFP mice and optogenetically stimulated ChAT-cre mice. For water and saccharine control experiment, 0.3% saccharine solution was made and consumption measured during the same optogenetic stimulation protocol.

### Locomotor activity

Mice were handled every day for 2 weeks prior to initiation of the experiment. Locomotor activity was measured in ChAT-cre and ChAT-ChR2-eYFP mice using a cage-rack photobeam system (PAS, San Diego Instruments) and the corresponding PAS software. Mice were placed in a novel cage within the locomotor apparatus, and ambulation (locomotion) was measured as the breaking of two distinct beams 10 cm apart. Locomotor activity was recorded for 40 min following 15 min of habituation.

### Analysis

Results are reported as mean ± SEM. Specific statistical tests used are detailed within each figure legend. Comparisons between two experimental conditions were made using Student’s paired *t*-test and K-S test for cumulative probabilities using Prism 7.0 (GraphPad Prism, San Diego, CA, USA). Electrophysiological and behavioral data of >2 groups were analyzed with Prism 7.0 (GraphPad Prism, San Diego, CA, USA) Statistics package using either one-way ANOVAs or with mixed-effects general linear model (SAS JMP 7.0) to account for random effect variables: animal ID, cell ID, antagonist treatment, and alcohol treatment. We used the EPHierStats approach to enable statistical comparisons of the frequencies distribution of interevent intervals (IEIs) where the traditional approach relies on a visual comparison combined with a Kolmogorov–Smirnov test that only assesses a single value in the distribution of frequencies. This novel approach also enables a full hierarchical general linear model to be used in this assessment which enables us to correct for covariates and test interactions with other independent variables. Subdividing measurements into biological replicates of 25 observations enables the selection of values corresponding exactly to the 6th, 26th, median, 74th, and 94th percentile values. These values were selected to represent the median, shoulders, and extreme values of the distribution we aimed to describe but could easily be replaced with other percentile values if appropriate. The inclusion of ten replicates of 25 measurements per condition enabled very powerful statistical comparisons but a lower number of replicate intervals will typically be sufficient to accurately quantify within-neuron variability. Selection of the five values per interval was accomplished here in MS Excel following organization of the dataset using the Sort command and can be accomplished easily in a wide range of software packages. Visual inspection of residuals of the various models confirmed that normalization using the ^40^√x transformation worked well for our dataset and enabled us to use parametric statistical tests. Proper encoding of the hierarchical relationships between the measurements from biological replicates is done using a mixed-effects general linear model in which (at a minimum) neuron and time interval should be included as random variables. The percentile [6, 26, 50, 74, 94] of each included value should always be included as a categorical fixed main effect in the statistical model as well as the interaction between the percentile and the factor of interest (i.e., percentile*genotype). If this interaction term does not substantially reduce the unexplained variance in the model, the interpretation of the statistical results might be simplified by removing this interaction term from the model.

## Results

**FIGURE 1:**
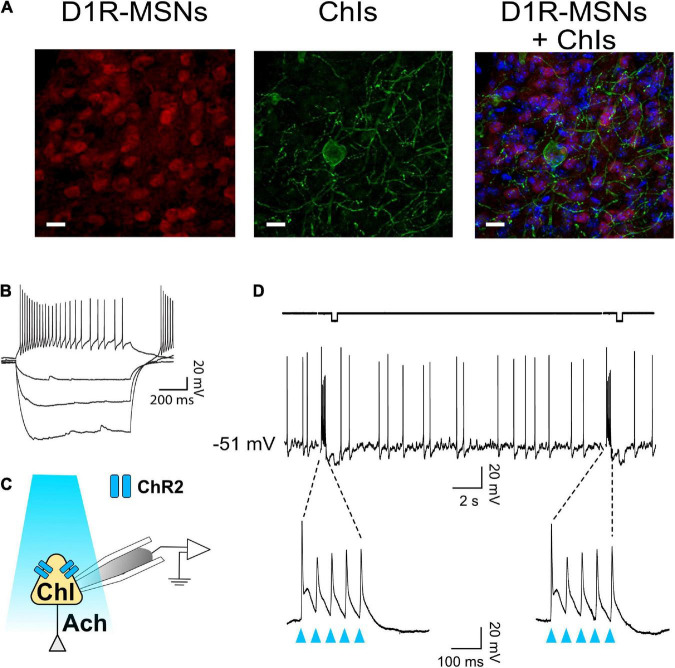
ChAT-ChR2-eYFP x DrD1-tdTomato mouse line to optogenetically stimulate ChIs and differentiate core NAc D1- and D2-MSNs. **(A)** Immunostaining in ChAT-ChR2-eYFP x DrD1-tdTomato mouse line of D1-MSNs (left panel, red-fluorescence) and cholinergic interneurons (middle panel, eYFP fluorescence) in the nucleus accumbens. Right panel shows overlaid left and middle panels. All neurons are stained in blue. Scale bar 20 um. **(B)** Representative voltage traces in response to incremental current steps (–150 pA to 0 in steps of 50) in a cholinergic interneuron. **(C)** Schematic of ChI recording during optogenetic stimulation with blue light. **(D)** Optogenetic stimulation (i.e., burst of five 20 Hz pulses every 20 s for 2 min) evokes action potentials in ChI.

ChAT.ChR2.eYFP and DrD1.TdTomato mouse lines were crossed to generate brain slices in which D1-MSNs could be identified while the role of ChIs in regulating glutamatergic synaptic transmission onto NAc MSNs could be assessed through optogenetic stimulation. Immunostaining showed the presence of eYFP and TdTomato reporters for ChIs and D1-MSNs, respectively, in the NAc (Figure 1A). eYFP-positive neurons were confirmed to be ChIs by injecting incremental current steps and recording voltage responses: current-voltage relationships presented the hallmarks of cholinergic interneurons, i.e., depolarized resting membrane potential (∼−50 mV), large membrane resistance and sag, and spontaneous firing (Figure 1B). To verify that ChIs expressed functional Channelrhodopsin (Figure 1C), ChIs were stimulated with blue light (five light pulses at 20 Hz every 20 s for 2 min). This pattern faithfully evoked action potentials in all (*n* = 8) neurons tested (Figure 1D).

### Cholinergic interneurons decrease glutamate release in D1- and D2-medium spiny neurons

**FIGURE 2:**
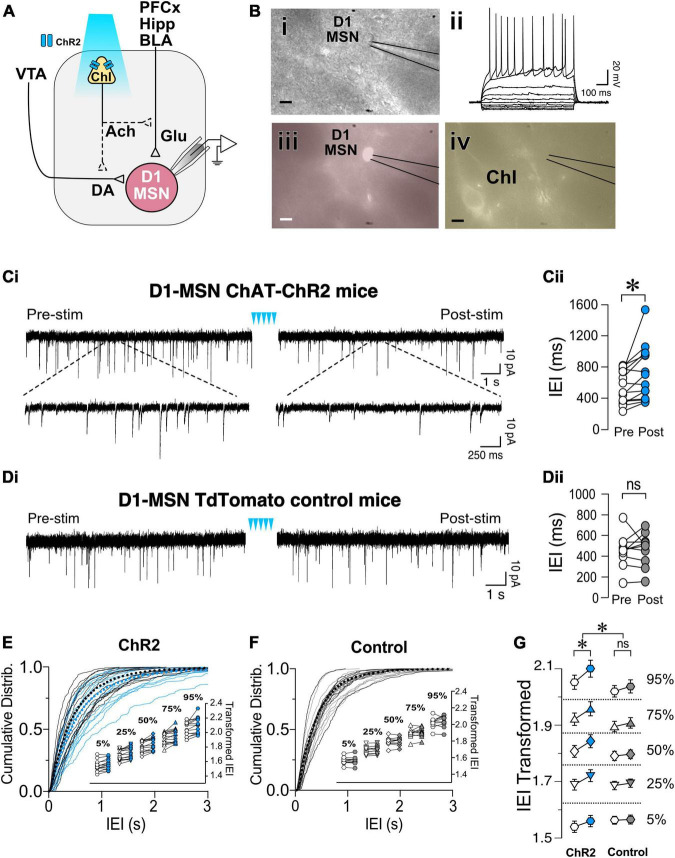
Optogenetic stimulation of ChIs decreases sEPSCs frequency in D1-MSNs. **(A)** Schematic of the experimental setup: whole cell recording of D1-MSNs sEPSC during optogenetic stimulation of ChIs. **(B)** Same slice images of representative MSNs [**(i)**, DIC], corresponding IV traces to confirm MSN identity **(ii)**, red-epifluorescence to verify the cell is D1R+ **(iii)**, and eYFP epifluorescence of ChI cell in proximity of the recording **(iv)**. Scale bar 10 um. **(Ci)** Representative sEPSCs in D1-MSNs in ChAT-ChR2 mice before (Pre) and after (Post) ChI optogenetic stimulation (blue arrowheads). **(Cii)** Average sEPSCs inter-event intervals (IEI) in Pre (white circles) and Post (blue circles) ChI optogenetic stimulation in ChAT-ChR2 D1-MSNs (*n* = 14). **(Di)** Representative traces of sEPSCs in D1-MSNs of tdTomato control mice before (Pre) and after (Post) ChI stimulation. **(Dii)** Average EPSCs inter-event intervals (IEI) in Pre (white circles) and Post (gray circles) ChI optogenetic stimulation in control tdTomato D1-MSNs (*n* = 10). **(E)** Cumulative frequency distribution of D1-MSN sEPSCs IEI in ChAT-ChR2 mice group Pre (black traces) and Post (blue traces) ChI optogenetic stimulation. Each solid line represents a neuron. Average traces for Pre and Post conditions are shown in dotted black and blues lines, respectively. Inset. Cumulative distributions of ChAT-ChR2 D1-MSNs EPSCs IEIs broken into percentiles of distribution to quantify median (50%), shoulders (25 and 75%), and extreme values (5 and 94%) of distribution, that are 1^10^ transformed to normalize the distribution. **(F)** Cumulative frequency distribution of D1-MSNs inter-event intervals (IEI) of sEPSCs before (Pre, black lines) and after (Post, gray lines) ChI optogenetic stimulation in tdTomato control mice. Each solid line represents a neuron. Average traces are shown in dotted black and blues lines for Pre and Post conditions, respectively. Inset. Same as inset in panel **(E)**, but in tdTomato D1-MSN controls. **(G)** Percentiles of cumulative distribution of transformed IEIs of EPSCs in ChAT-ChR2 D1-MSNs (Pre, white circles, Post, blue circles) and control TdTomato D1-MSNs (Pre, white circles, Post, gray circles). *p < 0.05, ns, no significant difference.

To determine whether ChIs controlled glutamate release in D1-MSNs, sEPSCs were recorded in TdTomato-labeled neurons while ChIs were simultaneously stimulated (Figure 2A) with a pattern described in Figure 1. Current-voltage relationships confirmed that all recorded red epifluorescent neurons (Figure 2Biii) were MSNs (Figures 2Bi,ii). Often, the cell body of ChIs could be detected in the vicinity of recorded MSNs (Figure 2Biv). sEPSCs were recorded at MSNs’ resting membrane potential (−85 ± 0.7 mV in a random sample of 10 neurons) for 4 min (Pre-stim; Figure 2Ci) before stimulating ChIs for 2 min (Figure 2Ci, blue arrowheads), followed by recording sEPSCs for 4 min (Post-stim; Figure 2Ci). The inter-event intervals (IEIs) between sEPSCs lengthened during the Post-stim vs. the Pre-stim interval [i.e., a decreased frequency; Figures 2Ci,ii, *t*(13) = 2.868, *p* = 0.0132, paired *t*-test, *n* = 14]. Interestingly, ChI stimulation did not affect the amplitude of D1-MSN sEPSCs in ChAT-ChR2 mice [Supplementary Figure 1, *t*(13) = 2.04, *p* = 0.0619, paired *t*-test, *n* = 14]. To verify that these effects are specifically due to optogenetic ChI stimulation, D1-MSN sEPSCs were recorded in slices obtained from the DrD1.Tdtomato mouse line (Figure 2Di). These recordings demonstrating that optical stimulation did not significantly affect IEIs [Figure 2Dii, *t*(9) = 0.2512, *p* = 0.8073, paired *t*-test, *n* = 10] or amplitude [Supplementary Figure 1, *t*(9) = 1.571, *p* = 0.1506, paired *t*-test, *n* = 10]. Comparisons of IEI cumulative frequency distributions revealed a significant difference between Pre- and Post-ChI stimulation conditions in D1-MSNs ChAT.ChR2 mice (Figure 2E, *D* = 0.07492, *p* < 0.0001, K-S test) but not in D1-MSN TdTomato control mice (Figure 2F, *D* = 0.03261, *p* = 0.0603, K-S test). Next, the relationship between sEPSCs’ IEI size and the ChI-mediated effect was assessed. To address this relationship, sets of 25 consecutive IEIs were ordered by IEI and the 50 (median), 25 and 75 (shoulders), as well as the 5 and 95 (extremes) percentile values were analyzed. No effect of different IEI size distribution on ChI-mediated IEI increase was observed [Figure 2E inset, *F*_(4,907)_ = 0.3362, *p* = 0.85, Mixed-model general linear modeling (MM GLM)], indicating that EPSCs are uniformly altered by ChIs. Finally, a significant difference in response to ChI stimulation was observed between ChR2 and TdTomato groups [Figure 2G; *F*_(1,2265)_ = 25.58, *p* < 0.0001, MM GLM, with Tukey *Post-hoc* tests revealing significant increases in IEI size between Pre- and Post-stimulation in ChR2 but not in TdTomato controls]. Interestingly, ChI stimulation had no effects on electrically evoked EPSPs in D1-MSNs [Supplementary Figure 1, *F*_(2,10)_ = 0.973, *p* = 0.3785, RM one-way ANOVA] and TdTomato control mice [Supplementary Figure 1, *F*_(2,6)_ = 1.7, *p* = 0.238, RM one-way ANOVA]. These results indicate that optogenetic stimulation of ChIs decreases only spontaneous glutamate release onto D1-MSNs, a likely presynaptic effect.

**FIGURE 3:**
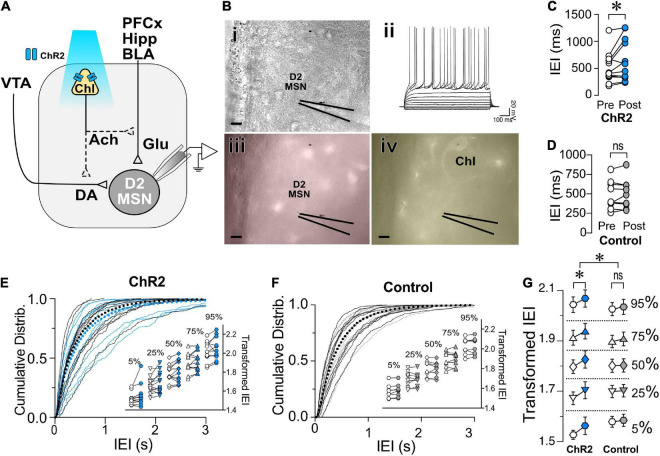
Optogenetic stimulation of ChIs decreases sEPSCs frequency in D2-MSNs. **(A)** Schematic of the experimental setup: whole cell sEPSC recording of D2-MSNs during optogenetic stimulation of ChIs. **(B)** Same slice images of representative MSNs [**(i)**, DIC], corresponding IV traces to confirm MSN identity **(ii)**, lack of red-epifluorescence to verify the cell is D1R (–) **(iii)**, and eYFP epifluorescence of ChI cell in the proximity of the recording (iv). Scale bar 10 um. **(C)** Average sEPSCs IEI before (Pre, white circles) and after (Post, blue circles) optogenetic stimulation of ChIs in ChAT-ChR2 D2-MSNs (*n* = 12). **(D)** Average sEPSCs IEI before (Pre, white circles) and after (Post, gray circles) optogenetic stimulation of ChIs in tdTomato control mice (*n* = 9). **(E)** Cumulative frequency distribution of D2-MSN IEI of sEPSCs in ChAT-ChR2 mice before (Pre, black traces) and after (Post, blue traces) ChI optogenetic stimulation. Each solid line represents a neuron. Average traces are shown in dotted black and blues lines for Pre and Post conditions, respectively. Inset. Cumulative distributions of ChAT-ChR2 D2-MSNs EPSCs IEIs broken into percentiles of distribution to quantify median (50%), shoulders (25 and 75%), and extreme values (5 and 94%) of distribution, that are 1^10^ transformed to normalize the distribution. **(F)** Cumulative frequency distribution of D2-MSN IEI of sEPSCs in tdTomato mice before (Pre, black traces) and after (Post, blue traces) ChI optogenetic stimulation. Each solid line represents a neuron. Average traces are shown in dotted black and blues lines for Pre and Post conditions, respectively. Inset. Same as inset in panel **(E)**, but in TdTomato D2-MSN controls. **(G)** Percentiles of cumulative distribution of transformed IEIs of EPSCs in ChAT-ChR2 D2-MSNs (Pre, white circles, Post, blue circles) and control TdTomato D2-MSNs (Pre, white circles, Post, gray circles). *p < 0.05, ns, no significant difference.

To determine whether ChIs similarly regulated glutamatergic synaptic transmission in putative D2-MSNs, non-fluorescent MSNs were recorded while stimulating ChIs optogenetically (Figures 3A,B). Current-voltage relationships confirmed that all recorded neurons were MSNs (Figure 3Bii). As with D1-MSNs, putative D2-MSNs’ baseline sEPSCs was recorded for 4 min before (Pre) and after (Post) optogenetic ChIs stimulation. Similar to D1-MSNs, blue light stimulation significantly increased average IEIs [i.e., decrease frequency; Figure 3C, *t*(11) = 2.27, *p* = 0.0443, paired *t*-test, *n* = 12] in sliced obtained from ChAT-ChR2 mice but not in TdTomato control slices [Figure 3D, *t*(8) = 0.604, *p* = 0.563, paired *t*-test, *n* = 9]. Likewise, significant increase of IEI sEPSCs cumulative frequency distribution in Pre vs. Post groups in D2-MSNs of ChAT-ChR2 mice was observed [Figure 3E, *D* = 0.048, *p* = 0.0011, K-S test, *n* = 12], but not in D2-MSNs of TdTomato control mice [Figure 3F, *D* = 0.0175, *p* = 0.7278, K-S test, *n* = 9]. As with D1-MSNs, significant increases of IEI were observed at all percentiles in D2-MSNs of ChAT-ChR2 (Figure 3E, inset), but not TdTomato control mice (Figure 3F, inset). Finally, a significant difference in response to ChI stimulation between ChR2 and TdTomato control groups was observed [Figure 3G; *F*_(1,1915)_ = 18.05, *p* < 0.0001, MM GLM, with Tukey *post-hoc* tests showing significant increase in IEI duration between Pre- and Post-intervals in ChR2 but not in TdTomato controls]. No effects of ChI stimulation on D2R MSNs sEPSC amplitude was observed in ChR2 [Supplementary Figure 2, *t*(11) = 0.129, *p* = 0.8995, paired *t*-test], or TdTomato control group [Supplementary Figure 2, *t*(8) = 0.277, *p* = 0.789, paired *t*-test]. Interestingly, electrically evoked EPSPs in D2-MSNs following ChI optogenetic stimulation had a significantly decreased amplitude in ChR2 groups [Supplementary Figure 2, *F*_(2,8)_ = 6.01, *p* = 0.0145], but not in TdTomato control groups [Supplementary Figure 2, *F*_(2,6)_ = 0.20, *p* = 0.742]. These results indicate that ChIs stimulation decreases glutamate release presynaptically onto D1- and D2-MSNs.

### Control of glutamate release by cholinergic interneurons in D1- and D2-medium spiny neurons involves different mechanisms

**FIGURE 4:**
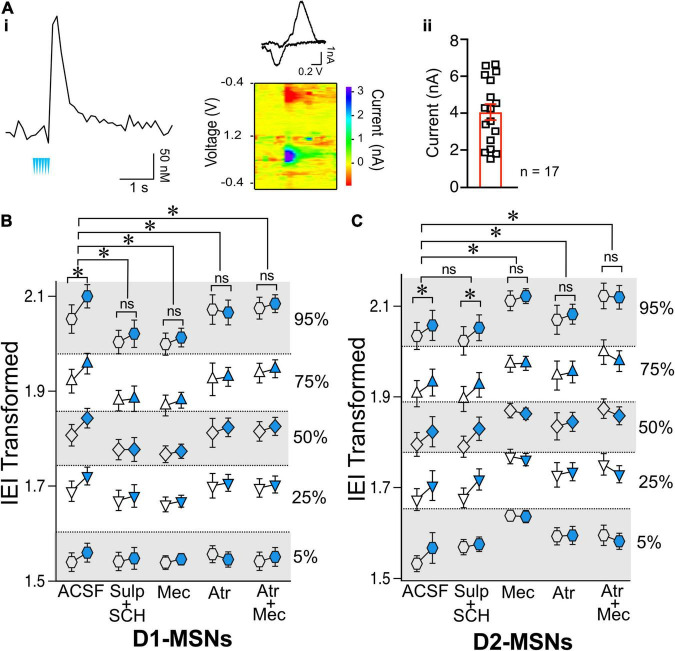
Effects of ChIs on D1- and D2-MSNs glutamate release are mediated through different pathways. **(A)** Optogenetic activation of ChIs evokes dopamine release in NAc as measured by voltammetry. **(i)** Representative DA trace and cyclic voltammogram showing characteristic DA waveform. **(ii)** DA responses evoked from ChI stimulation scatter plot and average ± SEM showing the range of DA currents from 6 slices and 17 recordings. **(B)** Transformed IEI of D1-MSNs sEPSCs shown as percentiles of cumulative distribution. Data for the control group (ACSF) is reproduced from Figure 2G. IEI is shown before (Pre, white circles) and after (Post, blue circles) ChIs stimulation in control conditions (ACSF) and in presence of antagonists: Sul + SCH (5 uM SCH-23390, D1 receptor antagonist + 1 uM sulpiride, D2 receptor antagonist), Atr (1 uM atropine, mAChR antagonist), Mec (5 uM mecamylamine, nAChR antagonist), and Atr + Mec (atropine + mecamylamine). **(C)** Same as panel **(B)** in D2-MSNs. *p < 0.05, ns, no significant difference.

Having shown that ChIs decrease the release of glutamate from presynaptic terminals synapsing on both D1- and D2-MSNs, we questioned whether these two neuronal populations shared the same mechanisms. There is strong evidence that ChIs induce DA release in the striatum (Cachope et al., 2012; Threlfell et al., 2012). Because DA regulates glutamate release (Wang et al., 2013), the hypothesis that ChIs’ effects on glutamate release involved DA was tested in ChAT.ChR2.eYFP x DrD1-TdTomato mice. First, we confirmed that optogenetic stimulation of ChIs indeed evoked DA release (Figures 4Ai,ii) measured with fast-scan cycling voltammetry (FSCV), when using the same stimulation pattern as that of our electrophysiological experiments. We then tested the putative role of DA in mediating ChIs-induced inhibition of glutamate release in D1-MSNs. ChI stimulation resulted in significantly longer IEIs in of D1-MSNs (Figure 4B, ACSF group as already presented in Figure 2G). In the presence of dopamine D1- and D2-receptor antagonists IEIs were no longer lengthened by optogenetic ChI stimulation [Figure 4B, ACSF vs. Sulp + SCH, *F*_(1,2514)_ = 32.12, *p* < 0.0001, MM GLM], with Tukey *post-hoc* tests revealing significant differences between Pre and Post conditions in ACSF condition, while the Pre- and Post-stimulation groups with dopamine antagonists were not significantly different. These findings show that the effect of ChI activity is DA-dependent in D1-MSNs with DA released by ChI stimulation likely acting on DA receptors expressed on glutamatergic terminals (Dumartin et al., 2007; Wang et al., 2012). Next, the dependency of the DA effect on nAChR activation was confirmed [Figure 4B, ACSF vs. Mec, *F*_(1,2562)_ = 28.25, *p* < 0.0001, MM GLM, with Tukey *post-hoc* tests showing no differences between Pre and Post conditions when nAChRs were blocked]. Antagonizing mAChR signaling also prevented the ChI-activation mediated increase in IEI [Figure 4B, ACSF vs. Atr, *F*_(1,2269)_ = 56.81, *p* < 0.0001, MM GLM, with Tukey *post-hoc* tests showing no differences between Atr Pre and Post conditions]. In line with the preceding outcomes, antagonists of both mAChR and nAChR also blocked the increase of IEIs [Figure 4B, ACSF vs. Atr + Mec, *F*_(1,2521)_ = 28.15, *p* < 0.0001, MM GLM, with Tukey *post-hoc* tests showing no differences between Atr + Mec Pre and Post conditions]. Finally, bath application of antagonists did not significantly change glutamate release under baseline conditions [Supplementary Figure 3, D1-MSNs, *F*_(4,60)_ = 1.472, *p* = 0.22, one-way ANOVA]. Together, these results indicate that mAChR, nAChR and DA receptor signaling are all required to mediate the effects of ChIs on glutamate transmission in D1-MSNs.

Next, the mechanisms underlying ChIs-mediated inhibition of glutamate release were assessed in D2-MSNs. Surprisingly, in D2-MSNs the effect of ChI stimulation on IEI length was not significantly different between the ACSF and DA receptor antagonists groups [Figure 4C, ACSF vs. Sul-SCH, *F*_(1,1821)_ = 0.2625, *p* = 0.6085, MM GLM], with ChI stimulation significantly lengthening IEI in both groups (*p* < 0.05). This suggested that ChIs exert a direct presynaptic control over glutamate release in D2-MSNs that does not require DA. Interestingly, blocking nAChR signaling in D2-MSNs prevented the ChI stimulation induced lengthening of IEI [Figure 4C, ACSF vs. Mec, *F*_(1,1804)_ = 25.34, *p* < 0.0001, MM GLM, with Tukey *post-hoc* tests showing no difference in Mec Pre and Post groups]. Recordings in the presence of the mAChR blocker atropine were also significantly different from ACSF control [Figure 4C, ACSF vs. Atr, *F*_(1,1753)_ = 3.537, *p* = 0.0402, MM GLM, with Tukey *post-hoc* tests revealing no difference in the Atr Pre and Post groups]. Accordingly, simultaneous application of both nAChR and mAChR antagonists found similar blockage of the ChI effect on IEI [Figure 4C, ACSF vs. Atr + Mec, *F*_(1,1760)_ = 55.05, *p* < 0.0001, MM GLM, with Tukey *post-hoc* tests showing no difference in Atr + Mec Pre vs. Post groups]. These findings indicate that, unlike in D1-MSNs, ChI-mediated decrease in glutamate release in D2-MSNs is DA-independent but still mediated by both mAChR and nAChR antagonists.

While muscarinic and DA receptors antagonists applied under baseline conditions (without optogenetic stimulation) did not alter sEPSCs IEIs (Supplementary Figure 3, D2-MSNs, Atr: *p* = 0.29, Atr + Mec: *p* = 0.11, Sul + SCH: *p* = 0.20), blocking nicotine receptors with mecamylamine significantly increased IEIs (Supplementary Figure 3, D2-MSNs Mec, Mann–Whiteny *U* = 21, *p* = 0.0387, Mann–Whitney test). This finding provides a possible explanation for the observation that blocking nicotinic receptor with mecamylamine in D2-MSNs increased IEI: bath application of mecamylamine significantly decreased glutamate release, thus reaching a “floor effect” that could not be further decreased by ChIs stimulation (Figure 4C), indicating high nicotinic receptor sensitivity to the baseline tonic ACh release.

### Binge alcohol drinking selectively reverses the effect of cholinergic interneuron-mediated glutamatergic synaptic transmission in D1-medium spiny neurons

**FIGURE 5:**
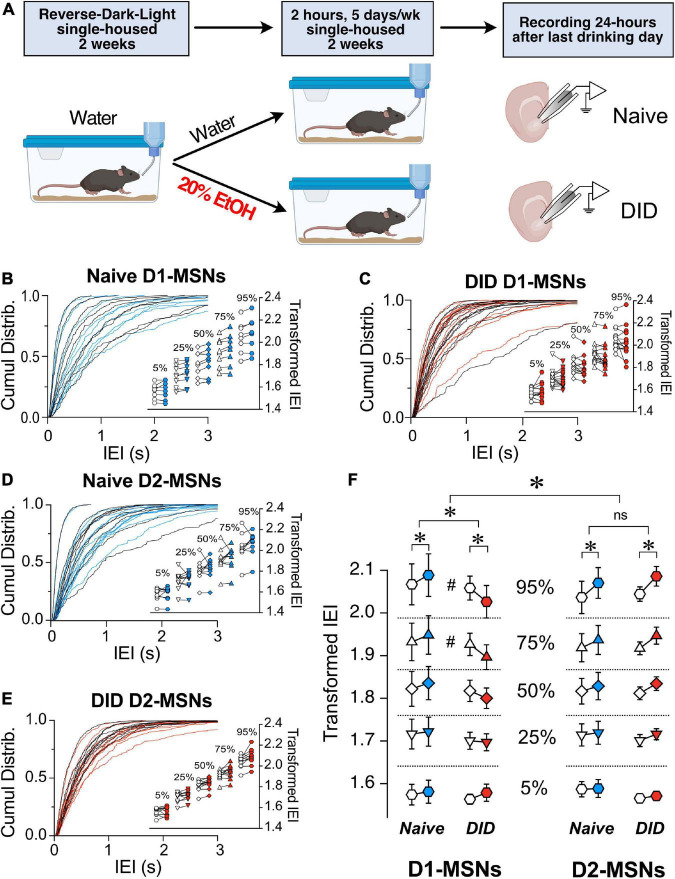
Binge alcohol drinking differentially affects the control by ChI on glutamate release in D1 and D2R MSNs. **(A)** Schematic of drinking in the dark (DID) treatment. Mice were single-housed at 4–5 weeks of age and placed into reversed dark-light schedule to habituate for 2 weeks and then given either water (Naïve group) or 20% EtOH (DID group) every day for 2 h, 5 days a week for 2 weeks. Brain slices were then isolated, and MSNs’ sEPSCs recorded. **(B)** Cumulative frequency distribution of D1-MSN IEI of sEPSCs in ChAT-ChR2 mice group before (Pre, black traces) and after (Post, blue traces) ChI optogenetic stimulation. Each solid line represents a neuron. Average traces for Pre and Post conditions are shown in dotted black and blues lines, respectively. Inset. Cumulative distributions of D1-MSNs EPSCs IEIs in ChAT-ChR2 mice broken into percentiles of distribution to quantify median (50%), shoulders (25 and 75%), and extreme values (5 and 94%) of distribution, that are 1^10^ transformed to normalize the distribution. **(C)** Same as panel **(B)** in DID mice. Black and red traces indicate IEI before and after ChI, respectively (*n* = 14). **(D)** Cumulative frequency distribution of D2-MSN IEI of sEPSCs in ChAT-ChR2 mice before (Pre, black traces) and after (Post, blue traces) ChI optogenetic stimulation. Each solid line represents a neuron. Average traces are shown in dotted black and blues lines for Pre and Post conditions, respectively. Inset. Cumulative distributions of ChAT-ChR2 D2-MSNs EPSCs IEIs broken into percentiles of distribution to quantify median (50%), shoulders (25 and 75%), and extreme values (5 and 94%) of distribution, that are 1^10^ transformed to normalize the distribution (*n* = 10). **(E)** Same as panel **(B)** in DID mice. Black and red traces indicate IEI before and after ChI, respectively (*n* = 11). **(F)** Percentiles of cumulative distribution of transformed IEIs of EPSCs in Naïve ChAT-ChR2 (Pre, white circles, Post, blue circles) and DID ChAT-ChR2 (Pre, white circles, Post, red circles) in D1- and D2-MSNs. *p < 0.05, #*p* < 0.05, ns, no significant difference.

The effects of preceding alcohol exposure on the ChI control of MSN synaptic excitability was assessed using the drinking-in-the-dark (DID) paradigm based on a well-established model of binge alcohol drinking (Rhodes et al., 2005). The DID paradigm allows mice to drink 20% alcohol for 2 h starting 2 h into the dark phase for 5 consecutive days per week (Figure 5A). After 2 weeks of drinking either 20% alcohol (DID group) or water (Naïve group), sEPSCs were recorded in D1- and D2-MSNs before and after optogenetic stimulation. As previously shown in Figures 2, 3, we constructed cumulative distribution plots for D1-MSNs (Naïve, Figure 5B and DID Figure 5C) and D2-MSNs (Naïve, Figure 5D and DID Figure 5E). Surprisingly, unlike in Naïve conditions, the effect of alcohol exposure on ChI regulation of sEPSC IEI length was significantly different in D1- and D2-MSNs [Figure 5F, *F*_(1,4778)_ = 12.08, *p* = 0.0005, MM GLM, 3-way interaction between optogenetic treatment, alcohol treatment and cell type]. Tukey *post-hoc* tests revealed that in naïve D1-MSNs, ChI activation increased IEI while in DID exposed D1-MSNs, IEIs were significantly decreased, thus reversing the ChI effect on glutamatergic transmission. Conversely, ChI activation resulted in longer IEIs in D2-MSNs of both naïve and DID exposed mice. Interestingly, the effects of alcohol treatment seen in D1-MSNs depended on the size of IEI [Figure 5F, D1-MSNs, *F*_(4,2374)_ = 3.900, *p* = 0.0037], with *post-hoc* tests revealing differences in the 75 and 95 percentiles, but not other intervals, indicating that alcohol effect was especially pronounced in largest size IEIs. In D2-MSNs there was no relationship between IEI size the effect of alcohol. These results demonstrate that preceding alcohol exposure selectively inverts the effect of ChI activation on D1-MSNs from reducing to increasing glutamate release onto D1-MSNs, while not having this effect on D2-MSNs. There was no significant difference in DID D1-MSNs sEPSC amplitude [Supplementary Figure 4, *t*(13) = 1.46, *p* = 0.1680, paired *t*-test] or in DID D2-MSNs sEPSC amplitude [Supplementary Figure 4, *t*(10) = 0.6948, *p* = 0.503, paired *t*-test].

**FIGURE 6:**
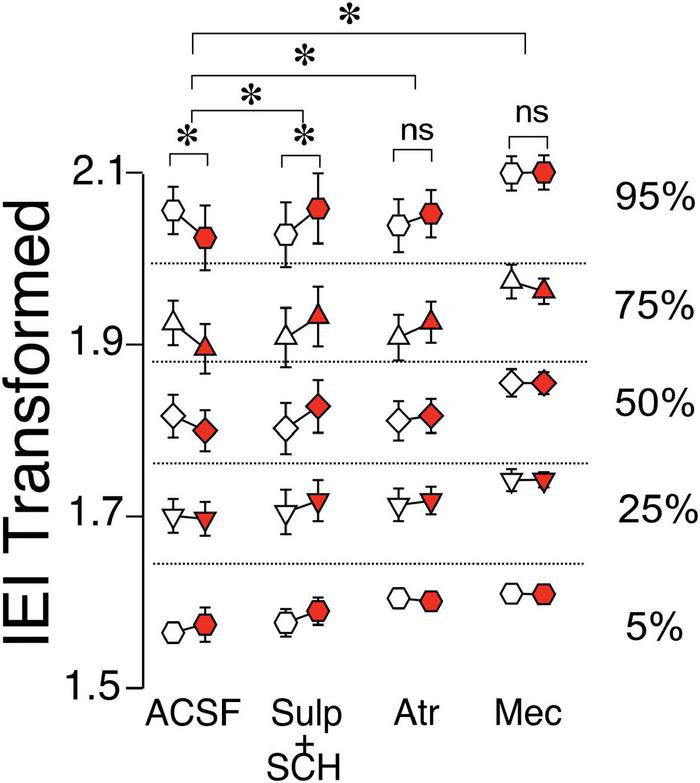
Effects of ChI stimulation in DID D1-MSNs in the presence of antagonists. IEI in DID D1-MSNs EPSCs shown as percentiles of cumulative distribution. Data for the control group (ACSF) is reproduced from Figure 5F. Each group of Pre (white circles) and Post (red circles) column is recorded either without (ACSF) or with the bath presence of antagonists: Sul + SCH (5 uM SCH-23390, D1 receptor antagonist + 1 uM sulpiride, D2 receptor antagonist), Atr (1 uM atropine, mAChR antagonist), Mec (5 uM mecamylamine, nAChR antagonist). *p < 0.05, ns, no significant difference.

Given the effects of preceding alcohol exposure on ChI regulation of glutamate release in D1-MSNs, the next objective was to identify receptors mediating these effects. Interestingly, in DID-exposed D1-MSNs, recording in the presence of D1- and D2-antagonists seemed to block the effect of DID [Figure 6, ACSF vs. Sulp + SCH, *F*_(1,2331)_ = 40.49, *p* < 0.0001, MM GLM]. This increase of IEI length following ChI stimulation in the presence of dopamine receptor antagonists in DID-exposed D1-MSNs was reminiscent of the naïve D1-MSN group (Figure 5F), indicating an important role of DA receptors in alcohol’s effect on ChI-modulated glutamate release. In the presence of mAChR antagonist atropine, ChI effect of sEPSC IEI was also significantly different from ACSF condition [Figure 6, ACSF vs. Atr, *F*_(1,2495)_ = 25.1129, *p* < 0.0001, MM GLM, with Tukey *post-hoc* showing a significant IEI decrease only in ACSF group, but no significant difference between Pre vs. Post groups in the Atr group]. Finally, recording in the presence of nAChR antagonist mecamylamine also abolished the effect of ChIs, and was significantly different from ACSF [Figure 6, ACSF vs. Mec, *F*_(1,2577)_ = 6.281, *p* = 0.0123, MM GLM, with Tukey *Post-hoc* similarly showing only a significant difference in the ACSF group]. The most parsimonious interpretation of these results is that the influence of preceding alcohol exposure on the ChI-mediated glutamate release in D1-MSNs mostly depends on dopaminergic signaling with additional influences from both nAChR and mAChR signals.

**FIGURE 7:**
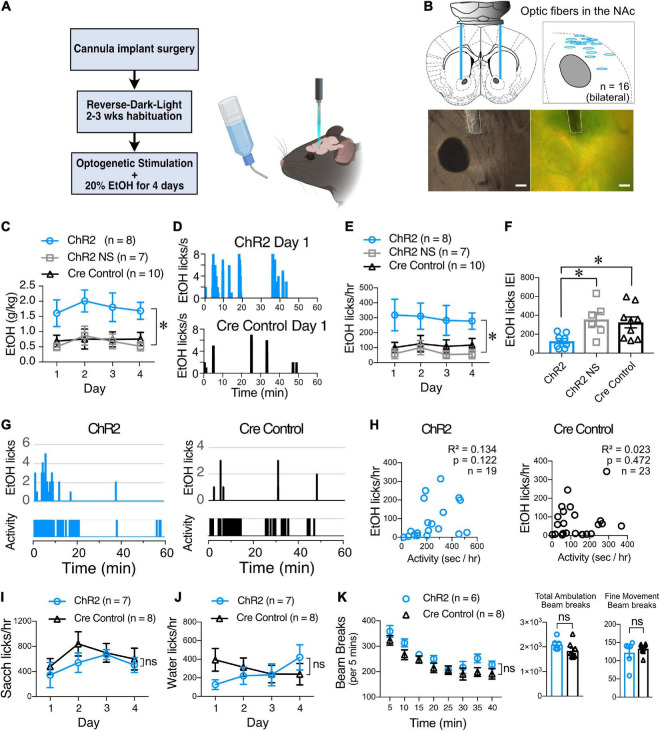
ChI optogenetic stimulation increases EtOH consumption. **(A)** Schematic of the DID behavioral experiment. ChAT.ChR2 and ChAT.cre mice underwent fiber optic implant in the NAc surgery at 4–5 weeks of age, recovered and habituated to reverse-dark-light schedule. ChAT.ChR2 and ChAT.cre mice were optogenetically stimulated during 4 days of 20% alcohol exposure and tethered to the fiber cord while ChAT.ChR2 not stimulated (NS) mice were only tethered to the fiber cord. **(B)** Schematic of the bilateral optic fiber cannula implant in the NAc (top left panel), locations of bilateral implants in 8 ChR2 mice that were used for the experiment (top right panel), example image of cannula placement in DIC (bottom left panel), and fluorescent light (bottom right panel). **(C)** Average daily alcohol consumption over 4 days in optically stimulated ChR2 mice (ChR2), non-stimulated ChR2 mice (ChR2 NS), and stimulated ChAT-cre mice (Cre controls). **(D)** Graphs of alcohol licks during 1 h period in representative stimulated (top graph, blue bars) and non-stimulated ChR2 mice (bottom, gray bars). **(E)** Daily average of alcohol licks over 4 days of alcohol exposure in optically stimulated ChR2 mice (ChR2), non-stimulated ChR2 mice (ChR2 NS), and stimulated ChAT-cre mice (Cre controls). **(F)** Frequency of alcohol licks as measured by average licking bout inter-event interval (IEI) per mouse over a 4-day period. **(G)** Representative plots of mouse activity as measured by passive infrared (PIR) activity monitoring system and corresponding EtOH licks in ChR2 (Left graphs, blue bars) and Cre control mice (right graphs, black bars). **(H)** The number of licks is not correlated with mice locomotor activity in ChR2 (blue symbols) and Cre control groups (black symbols). **(I)** Saccharine consumption measured as number of licks/hr over 4 days in 1 h-long optogenetically stimulated ChR2 (blue circles) and Cre control mice (black triangles). **(J)** Water consumption measured as number of licks/hr over 4 days in 1 h-long optogenetically stimulated ChR2 and Cre control mice. **(K)** Ambulatory activity test of ChR2 and Cre control mice during ChI optogenetic stimulation. Ambulation time course shows average beam breaks every 5 min for 40 min of the test. Total ambulation shows the total beam breaks in 40 min, fine movement shows grooming activity and vertical ambulation shows rearing activity. *p < 0.05, ns, no significant difference.

Interestingly, the application of bath antagonists in DID D1-MSNs changed only during mecamylamine treatment compared to ACSF (Supplementary Figure 5, Mec, Mann–Whiteny *U* = 22, *p* = 0.0065), while the other antagonists were not different from ACSF group (Supplementary Figure 5, Atr: p = 0.693, Sul + SCH: *p* = 0.896). This is in stark contrast from naïve D1-MSN group (Figure 4B), where mecamylamine treatment did not change the baseline, but is reminiscent of naïve D2-MSN group (Figure 4C), where mecamylamine also increased IEIs.

### Cholinergic interneuron optogenetic stimulation *in vivo* in the nucleus accumbens increases alcohol consumption in mice

Since binge alcohol drinking modulates the ChI-mediated synaptic transmission onto MSNs *ex vivo*, we next tested whether optogenetic stimulation of ChIs in freely-moving animals altered alcohol consumption. Fiber optic cannulas were implanted into the NAc of 4–5 weeks old mice that were allowed to recover and habituate to our reverse-light-dark room for 3 weeks before being optogenetically stimulated during the first 4 days of alcohol exposure (Figures 7A,B). From the very first day, the volume of alcohol consumed by stimulated ChAT.ChR2 mice group (Figure 7C, ChR2) was markedly larger compared to mice in the non-stimulated ChR2 group (Figure 7C, ChR2 NS) and stimulated ChAT.Cre controls [Figure 7C, *F*_(2,22)_ = 7.685, *p* = 0.0029, MM GLM, with Tukey *post-hoc* tests showing ChR2 group significantly different from both control groups]. The pattern of alcohol consumption was determined using lickometers by measuring the number and timing of licks of the drinking spout delivering alcohol (Figure 7D). The total number of licks in stimulated ChR2 mice during the 4 days was significantly increased compared to the non-stimulated and Cre control groups [Figure 7E, *F*_(2,22)_ = 6.50, *p* = 0.0061, MM GLM]. The increase in alcohol drinking was likely due to the increased frequency of consumption measured as the licking bout IEI was dramatically reduced in the ChR2 group [Figure 7F, *F*_(2,20)_ = 5.376, *p* = 0.0135, one-way ANOVA], and licks were highly correlated with the amount of alcohol consumed (Supplementary Figure 6). General locomotor activity of a subgroup of mice was measured using a passive infrared activity monitoring system and compared to alcohol licks during the same time interval (Figures 7G,H). This comparison illustrated that the increased drinking in the stimulated ChR2 mice could not be explained through a general increase in activity levels in these mice [Figure 7H; ChR2: *R*^2^ = 0.134, *p* = 0.122; Cre controls: *R*^2^ = 0.023, *p* = 0.472]. The increased alcohol consumption observed in stimulated ChR2 mice was specific to alcohol consumption and did not extend to the consumption of saccharine [Figure 7I, *F*_(1,47)_ = 1.53, *p* = 0.22, RM two-way ANOVA, Supplementary Figure 7, *F*_(1,47)_ = 1.78, *p* = 0.93, RM two-way ANOVA], and water [Figure 7J, *F*_(1,45)_ = 0.33, *p* = 0.57, RM two-way ANOVA, Supplementary Figure 7, *F*_(1,49)_ = 0.03, *p* = 0.87, RM two-way ANOVA]. Finally, stimulated ChR2 and Cre control mice (Figure 7K) did not differ in ambulation time course [*F*_(7,84)_ = 0.80, *p* = 0.59, RM two-way ANOVA], total ambulation [*t*(12) = 1.63, *p* = 0.13, student’s *t*-test], fine movement [*t*(12) = 1.12, *p* = 0.28, student’s *t*-test], or vertical motion [rearing, Supplementary Figure 7, *t*(12) = 0.8, *p* = 0.44, student’s *t*-test]. These results demonstrate that optogenetic stimulation of ChIs specifically altered alcohol consumption, without affecting water and saccharine drinking, an effect that was not due to increased activity levels.

## Discussion

**FIGURE 8:**
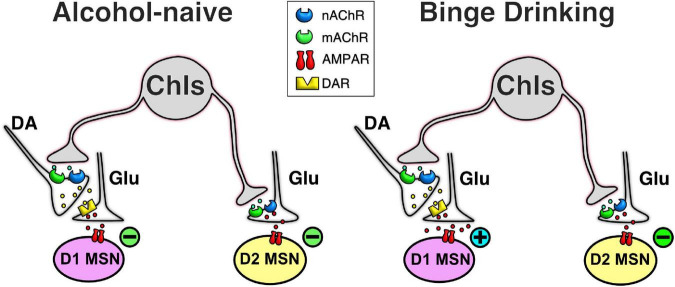
Simplified schematic of ChI effect on glutamatergic neurotransmission in MSNs in naïve and alcohol binge drinking mice. In D1-MSNs of naïve mice, ChIs decrease glutamatergic neurotransmission presynaptically through the release of DA in D1-MSNs **(top left cartoon)**, an effect that is reversed in DID mice **(top right cartoon)**. In contrast, in D2-MSNs of naïve mice, ChIs directly control glutamate release from terminals synapsing on D2-MSNs **(bottom left cartoon)**, an effect that is unchanged in DID mice **(bottom right cartoon)**.

The output neurons of the NAc, D1- and D2-MSNs, are a key part of the neurobiological mechanisms underlying drug addiction (Soares-Cunha et al., 2020) and altering their outputs will likely be an important part of any future treatments of alcohol addiction (Cheng et al., 2017). ChIs provide a promising avenue to do so since although these cells make up only 1–2% of the NAc neuronal population, they fulfill a key integrative role modulating the activity of MSNs (Kravitz et al., 2012). The data presented here show that ChIs control glutamatergic synaptic transmission in both D1- and D2-MSNs in alcohol-naïve mice, though the underlying regulatory mechanisms differ (Naïve, Figure 8). While the ChIs-driven decrease of glutamate release onto D1-MSNs is mediated by nicotinic and muscarinic ACh receptors through DA receptors, ChIs control of glutamate release onto D2-MSNs likely stems from ChIs directly synapsing on glutamatergic terminals through nicotinic and muscarinic ACh signals. Alternatively, ChIs’ effects could be mediated through the release of serotonin from raphe projections. Surprisingly, preceding alcohol exposure results in a switch where the effect of ChIs activity inverts from inhibiting to potentiating glutamatergic transmission in D1-MSNs while their inhibitory effect in D2-MSNs remains unchanged (Figure 8). Based on this dramatic change of its influence on D1-MSNs we hypothesized that altering ChI activity could be used to modulate alcohol drinking behavior. In line with this hypothesis, ChI optogenetic stimulation *in vivo* increased alcohol consumption in mice without altering locomotor activity, saccharine, or water consumption. Although, our study is limited to males, these findings identify NAc ChIs as key modulators of D1- and D2-MSNs synaptic excitability and suggest this cell population as a promising target of future addiction treatment strategies.

### Cholinergic interneurons decrease glutamate release in D1- and D2-medium spiny neurons through different mechanisms

Our finding that ChIs inhibit MSN glutamatergic synaptic transmission through a presynaptic mechanism confirms previous reports showing that acetylcholine receptor (AChR) agonists reduce the probability of glutamate release in the striatum (Malenka and Kocsis, 1988; Barral et al., 1999; Higley et al., 2009; Licheri et al., 2018). Similarly, direct stimulation of ChIs depressed electrically evoked EPSCs, an effect also attributed to presynaptic cholinergic receptors (Pakhotin and Bracci, 2007; Lee et al., 2016). Although MSNs express muscarinic M1 and M4 receptors (Hersch et al., 1994; Hersch and Levey, 1995), these receptors modulate voltage-not ligand-gated ion channels (Calabresi et al., 2000; Ebihara et al., 2013; Lv et al., 2017). Despite decades of research striving to understand how ChIs regulate glutamatergic synaptic transmission in MSNs, the mechanisms mediating their effects on D1- and D2-MSNs glutamatergic synaptic transmission is still poorly understood (Joshua et al., 2008; Cox and Witten, 2019). Our study provides evidence that ChIs employ different mechanisms to regulate glutamate release in D1- and D2-MSNs. Specifically, our data demonstrates the influence of DA on ChIs’ regulation of glutamate release in D1- but not D2-MSNs. The role of DA is supported by data from several groups showing that ChIs evokes DA release (Cachope et al., 2012; Threlfell et al., 2012; Wang et al., 2014; Shin et al., 2015), likely through α*β2 nAChR expressed on DA terminals (Threlfell et al., 2012; Yorgason et al., 2017). In addition to nAChRs, we found that mAChRs also contribute to the ChIs-mediated decrease of glutamate release in D1-MSNs, possibly through M5 mAChR (Grilli et al., 2008; Bendor et al., 2010; Kuroiwa et al., 2012; Shin et al., 2015), a finding confirming previous studies (de Rover et al., 2002; Ding et al., 2010; Pancani et al., 2014). Our study also indicates that, upon its release, DA binds to DA receptors located presynaptically on glutamatergic terminals where they decrease glutamate neurotransmission, a finding supported by several studies (Nicola et al., 1996, 2000; Nicola and Malenka, 1997; Dumartin et al., 2007), likely by promoting adenosine efflux *via* A1 adenosine receptors (A1Rs) (Harvey and Lacey, 1997; Ciruela et al., 2006). While our study confirms the role of DA and ACh receptors in regulating glutamatergic synaptic transmission, it provides key additional information as to how these neurotransmitters work together to regulate glutamate release in D1-MSNs.

As opposed to D1-MSNs, our data supports the notion that DA receptors do not contribute to ChI-mediated decrease of glutamate release in D2-MSNs. Instead, ChIs appear to send direct projections to glutamatergic terminals synapsing on D2-MSNs, an effect that our pharmacological experiments suggest is mediated by mAChRs. Although performed in conditions that did not differentiate D1- from D2-MSNs, several groups reported a similar contribution of mAChRs on glutamate release in the striatum (Calabresi et al., 2000; Higley et al., 2009; Ding et al., 2010; Pancani et al., 2014). Specifically, M2-4 mAChR located presynaptically on glutamatergic terminals directly decrease glutamate release by inhibition of P/Q-type VGCC and reduction of action potential–induced Ca^2+^ increases in the bouton (Calabresi et al., 2000; Higley et al., 2009; Pancani et al., 2014). We have also found that application of 1 μM nicotinic antagonist mecamylamine decreases sEPSCs in D2-MSNs. However, much ambiguity still exists on the nicotinic effect on glutamate release. α4β2 nAChR antagonist has been shown to increase glutamate release (Howe and Surmeier, 1995), while 1 and 10 μM nicotine application was shown to decrease sEPSCs frequency (Licheri et al., 2018), and 2 μM nicotine was also found to not change sEPSCs frequency (de Rover et al., 2002). Finally, we have found that ChIs inhibited, albeit moderately (i.e., <10%), electrically evoked EPSPs in D2-MSNs only. This result mirrors a similarly small reduction of EPSCs in unidentified MSNs reported by Pakhotin and Bracci (2007), an effect they attributed to a presynaptic action of Ach on glutamate release. Although muscarinic M1 and M4 receptors are also expressed in MSNs (Hersch et al., 1994), they appear to modulate MSNs intrinsic membrane properties (Ebihara et al., 2013; Lv et al., 2017), not synaptic transmission. Regarding interactions between dopamine and glutamatergic neurotransmission in the NAc, early studies observed a reduction of EPSP amplitudes, an effect attributed to a presynaptic D1Rs in glutamatergic terminals (Pennartz et al., 1992; Nicola et al., 1996; Harvey and Lacey, 1997; Nicola and Malenka, 1997). Although it is unclear why ChIs-induced DA release failed to inhibit evoked EPSPs in D1-MSNs in our study is unclear, it may simply reflect the vastly different experimental conditions between those used in the present study (i.e., optogenetics) and those used in early work (i.e., electrical stimulation). Nevertheless, these results emphasize the importance of distinguishing between striatal D1- and D2-MSNs when assessing their function in basal ganglia function.

### Alcohol exposure changes cholinergic interneuron control of the D1/D2 medium spiny neurons output balance

Unlike other drugs of abuse, alcohol does not have a single receptor, making identifying its targets difficult. Acute alcohol exposure modulates striatal output through ChIs (Adermark et al., 2011) and inhibits ChIs firing (Blomeley et al., 2011), while chronic alcohol use reduces density of cholinergic varicosities (Pereira et al., 2014). We found that ChIs’ stimulation increases alcohol consumption *in vivo*, while 2-week alcohol administration reverses the ChI control of glutamate release in D1-MSNs from inhibition to potentiation. Our finding is in line with previous studies showing that repeated exposure to alcohol potentiated D1-MSNs glutamatergic transmission (Cheng et al., 2017; Ji et al., 2017; Kircher et al., 2019; Strong et al., 2020). In addition to increasing glutamate release from terminals synapsing on D1-MSNs, chronic alcohol exposure was shown to act postsynaptically by increasing of spines density in dendrites of NAc and dorsal striatum MSNs (Nestby et al., 1999; Uys et al., 2015; Laguesse et al., 2018). Interestingly, glutamatergic transmission in D2-MSNs was not affected in binge alcohol drinking mice. Although this finding is somewhat surprising, Cheng et al. (2017) reported that chronic alcohol exposure did not alter evoked EPSCs amplitude but increased GABAergic neurotransmission in dorsal striatal D2-MSNs. Although we can only speculate about the specific origin of NAc D2-MSNs inhibitory inputs, it is worth noting that MSNs are mostly inhibited by GABAergic interneurons that are under the control of ChIs (de Rover et al., 2002; Witten et al., 2010). If true, this would strengthen the putative central role that ChIs play in regulating synaptic excitability of D1- and D2-MSNs through glutamatergic and GABAergic synaptic transmission, respectively, and in shaping the overall message sent to downstream brain regions. Although our behavioral data cannot fully account for the complex interactions between ChIs and MSNs glutamatergic synaptic transmission revealed by our electrophysiological data, they provide evidence that ChIs play a role in controlling alcohol consumption.

The mechanism responsible for reversing ChIs-mediated inhibition of glutamate release in D1-MSNs is unclear. Because DA is responsible for the ChIs-mediated decrease of glutamate release, increase of frequency observed in DID mice may result from alcohol either decreasing DA release (Karkhanis et al., 2015) and/or impairing nAChR (Hillmer et al., 2014) and mAChRs function (Costa and Guizzetti, 1999). Taken together, our findings offer a putative mechanism explaining why nAChR antagonists decrease alcohol consumption when administered i.p. (Ericson et al., 2009; Hendrickson et al., 2009, 2010), as well as directly into the NAc (Feduccia et al., 2014).

In summary, our study delineates a new understanding of the NAc circuitry and its effect on alcohol drinking behavior. ChIs likely induce DA release, which drives further alcohol consumption. Since ChI activation is what mediates this DA release, ChI stimulation *in vivo* will result in more DA released, driving the continuation of drinking after the very first sip (Beckley et al., 2016). On the other hand, after 2 weeks of daily alcohol exposure, ChIs preferentially and repeatedly stimulate D1-MSNs, which leads to disbalance between D1- and D2-MSNs, potentiating the D1-MSNs “go” direct pathway and inhibiting the M2-MSNs “no-go” indirect pathway. ChI-mediated reciprocal strengthening of “go” and inhibition of “no-go” pathways could be a core element of compulsive increase of drinking over time and transitioning to addiction (Koob et al., 1994; Kravitz et al., 2012). Therefore, inhibition of ChIs could be a future therapeutic target to treatment of alcohol use disorder.

## Data availability statement

The original contributions presented in this study are included in the article/Supplementary material, further inquiries can be directed to the corresponding author.

## Ethics statement

All mice were handled according to the American Association for the Accreditation of Laboratory Animal Care guideline. The protocol was approved by the Institutional Animal Care and Use Committee of University of Massachusetts Medical School.

## Author contributions

JK, PG-G, TL, and GM did the experiments. JK and VV analyzed data. JK, VV, and GM wrote the manuscript. All authors contributed to the article and approved the submitted version.

## Supplementary material

Supplementary Figure 1 D1-MSNs sEPSC and evoked EPSP measurements after ChI optogenetic stimulation. **(A)** Average sEPSCs amplitudes before (Pre, white circles) and after (Post, blue circles) ChI optogenetic stimulation in D1-MSNs ChAT.ChR2 mice (*n* = 14). **(B)** Average sEPSCs amplitudes before (Pre, white circles) and after (Post, gray circles) light stimulation of ChIs in D1-MSNs TdTomato mice (*n* = 10). **(C)** Electrically-evoked EPSPs in ChAT-ChR2 D1-MSNs before (Pre, white circles), during (white circles, blue bar), and after (Post, blue circles) ChI optogenetic stimulation (*n* = 10). **(D)** Electrically-evoked EPSPs in TdTomato control D1-MSNs before (Pre, white circles), during (white circles, blue bar), and after (Post, gray circles) ChI optogenetic stimulation (*n* = 7). **p* < 0.05.

Supplementary Figure 2 D2-MSNs sEPSC and evoked EPSP measurements after ChI optogenetic stimulation. **(A)** Average sEPSCs amplitudes before (Pre, white circles) and after (Post, blue circles) ChI optogenetic stimulation in D2-MSNs of ChAT.ChR2 mice (*n* = 11). **(B)** Average sEPSCs amplitudes before (Pre, white circles) and after (Post, gray circles) light stimulation in D2-MSNs of TdTomato mice (*n* = 9). **(C)** Electrically-evoked EPSPs in ChAT-ChR2 D2-MSNs before (Pre, black circles), during (Opto, white circles, blue bar), and after (Post, blue circles) ChI optogenetic stimulation (*n* = 9). **(D)** Same as panel **(C)** in tdTomato mice (*n* = 7). **p* < 0.05, ns, no significant difference.

Supplementary Figure 3 sEPSCs IEI in the absence and presence of ACh and DA antagonists in D1- and D2-MSNs in naive mice. Recordings of EPSCs IEIs during the bath application of antagonists. ACSF (control solution), D1 receptor antagonist Sulpiride + D2 receptor antagonist SCH-23390, nAChR antagonist mecamylamine, mAChR antagonist atropine. **p* < 0.05, ns, no significant difference.

Supplementary Figure 4 Binge alcohol drinking does not affect sEPSPs amplitude in D1- and D2-MSNs. **(A)** Amplitudes of DID D1-MSNs sEPSCs before (Pre, white circles) and after (Post, red circles) ChI optogenetic stimulation in ChAT.ChR2 mice (*n* = 14). Each symbol represents a MSN. **(B)** Amplitudes of DID D2-MSNs sEPSCs before (Pre, white circles) and after (Post, red circles) ChI stimulation in ChAT.ChR2 mice (*n* = 11). Each symbol represents a MSN. ns, no significant difference.

Supplementary Figure 5 sEPSCs IEIs in D1-MSNs in presence of ACh and DA receptors antagonists in DID mice. Average sEPSCs IEIs in DID D1-MSNs recorded in ACSF (control solution) and in presence of dopamine D1 and D2 receptor (Sul + SCH), mAChR (Atrop), and nAChR (Mec) antagonists groups. **p* < 0.05, ns, no significant difference.

Supplementary Figure 6 ChI optogenetic stimulation and EtOH consumption behavior. **(A)** Graph shows strong correlation between the volume of EtOH consumed and the number of licks in ChAT.ChR2 (ChR2) and ChAT.cre mice (Cre control). **(B)** 0.3% Saccharine consumed (g/kg) during 1-h long ChI optogenetic stimulation in ChAT.ChR2 and ChAT.cre mice. **(C)** Water consumed (g/kg) during 1 h-long ChI optogenetic stimulation in ChAT.ChR2 and ChAT.cre mice. **(D)** Vertical motion (rearing) in ChAT.ChR2 and ChAT.cre mice during ChI optogenetic stimulation. ns, no significant difference.

Supplementary Figure 7 Water and ethanol consumption in the binge alcohol drinking paradigm. **(A)** Amount of water consumed during the DID paradigm measured as milliliter of water consumed per mouse’s weight in kilograms. **(B)** Amount of 20% ethanol consumed during DID paradigm measured as grams of ethanol per mouse’s weight in kilograms (*N* = 8; Mean ± SEM).
