# Supplementary figures and images for: Binge alcohol drinking alters the differential control of cholinergic interneurons over nucleus accumbens D1 and D2 medium spiny neurons
Source: Front Cell Neurosci. 2022 Dec 15;16:1010121. doi: 10.3389/fncel.2022.1010121 (PMC9797504; doi:10.3389/fncel.2022.1010121)

**Suppl Fig. 1**

**
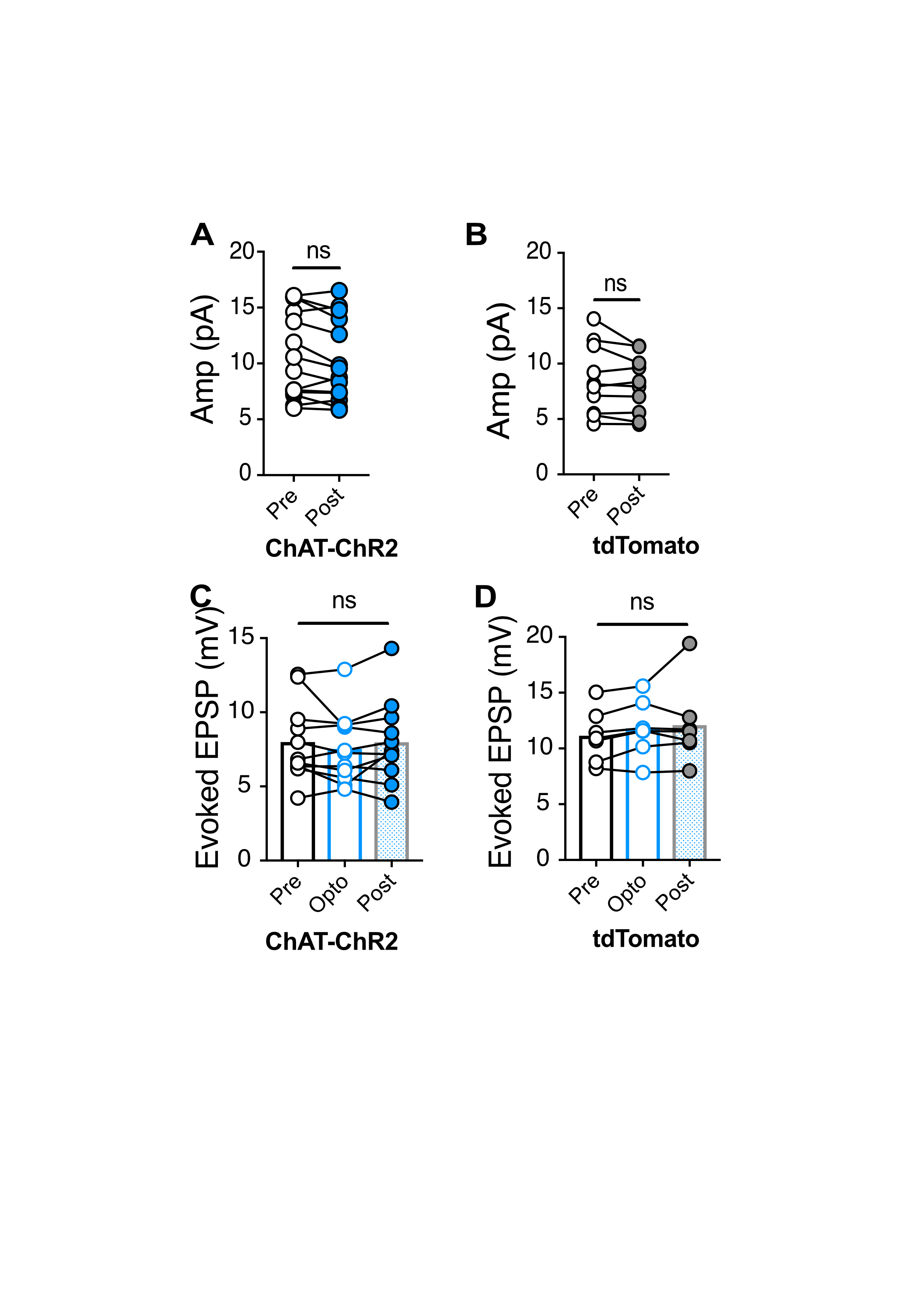
**

**Suppl Fig. 2**

**
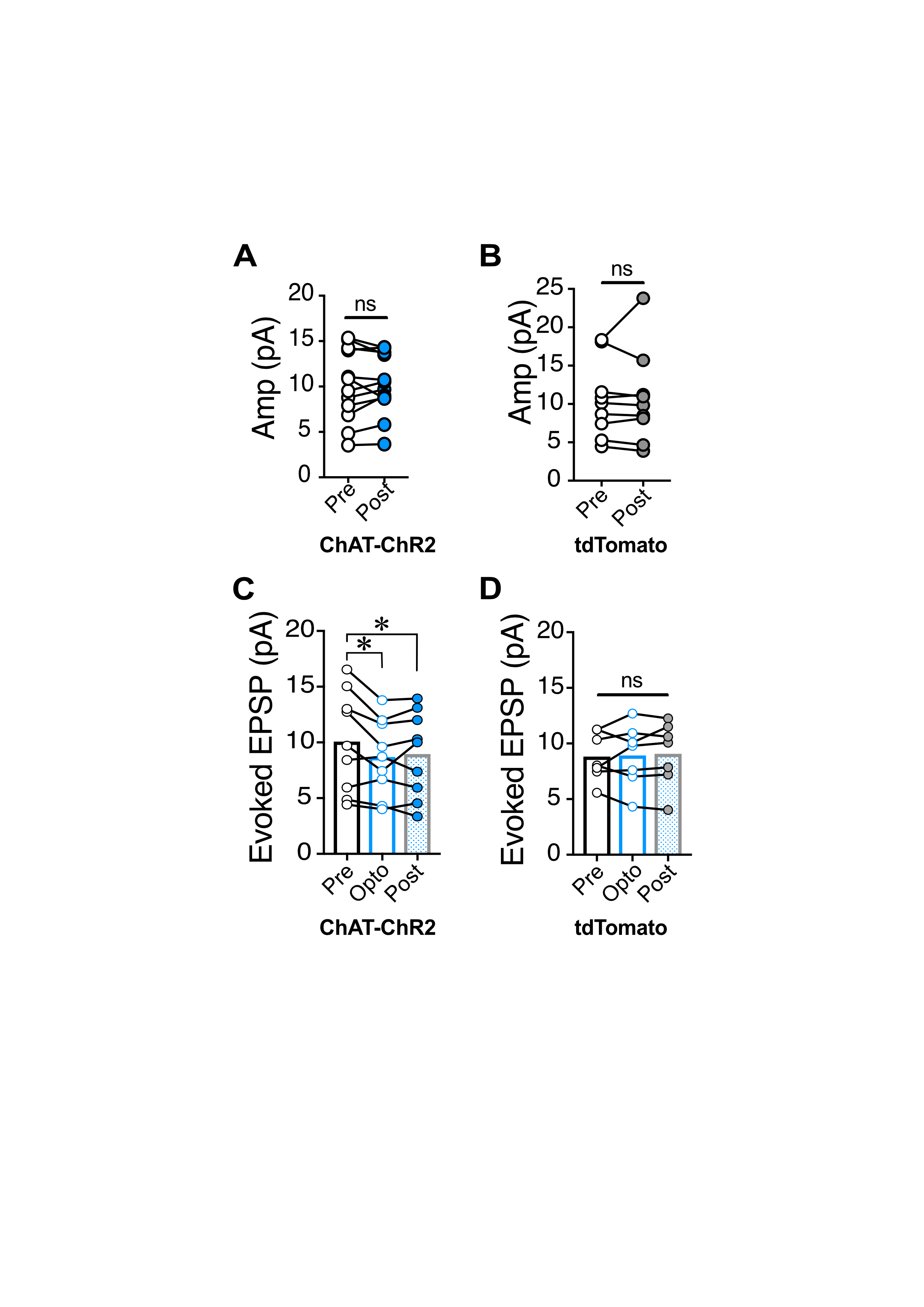
**

**Suppl Fig. 3**

**
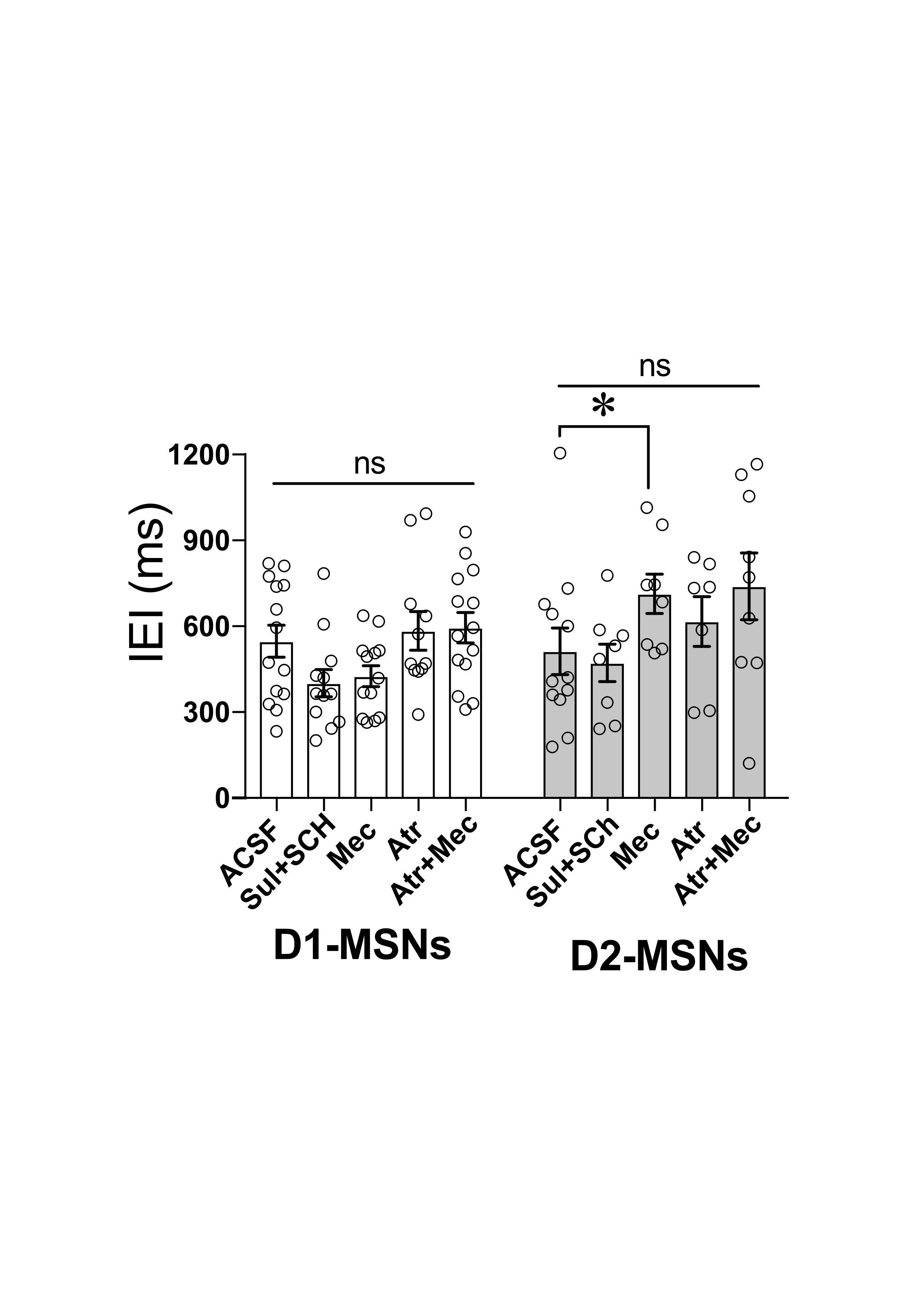
**

**Suppl Fig. 4**

**
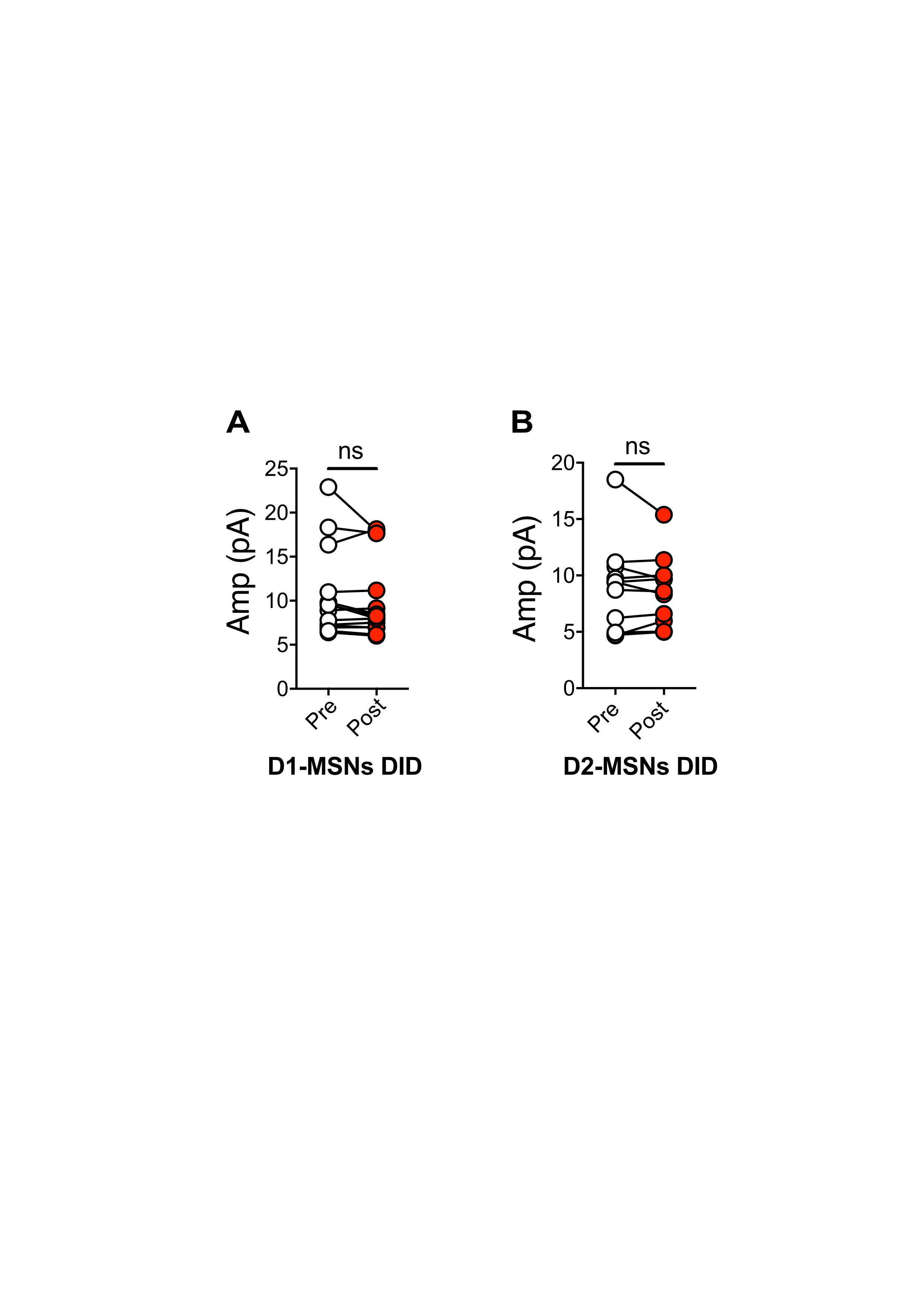
**

**Suppl Fig. 5**

**
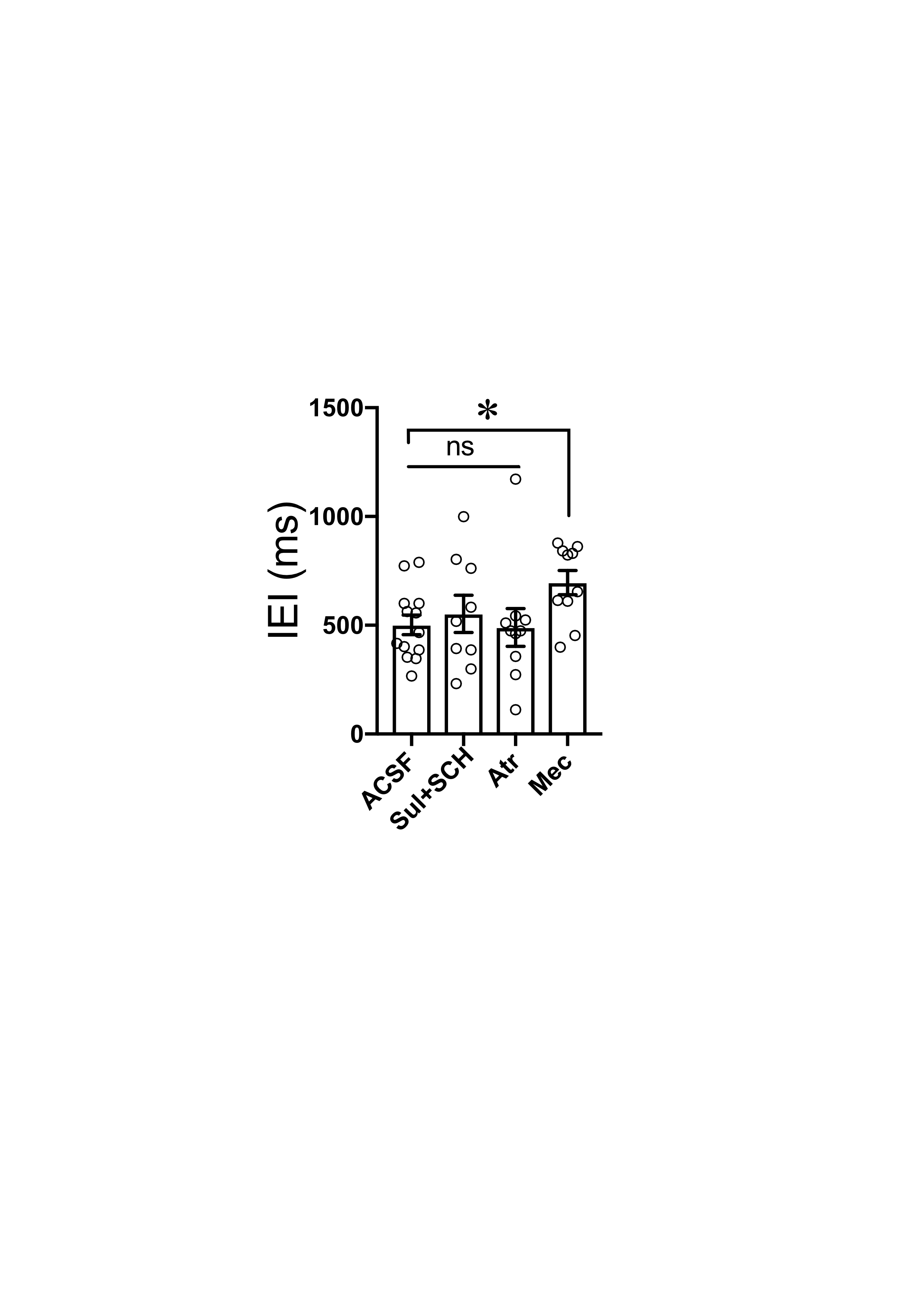
**

**Suppl Fig. 6**

**
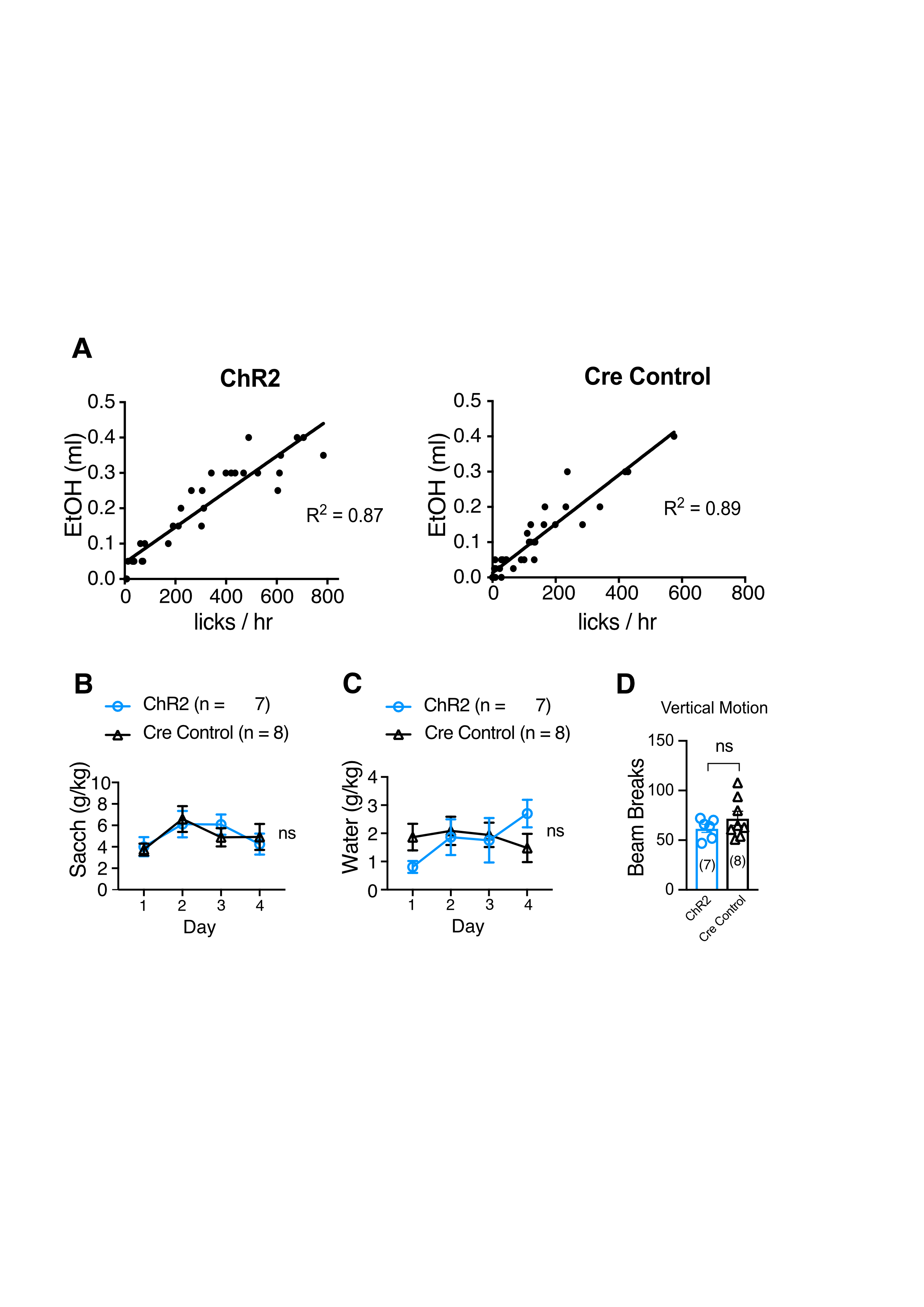
**

**Fig. 7**

**
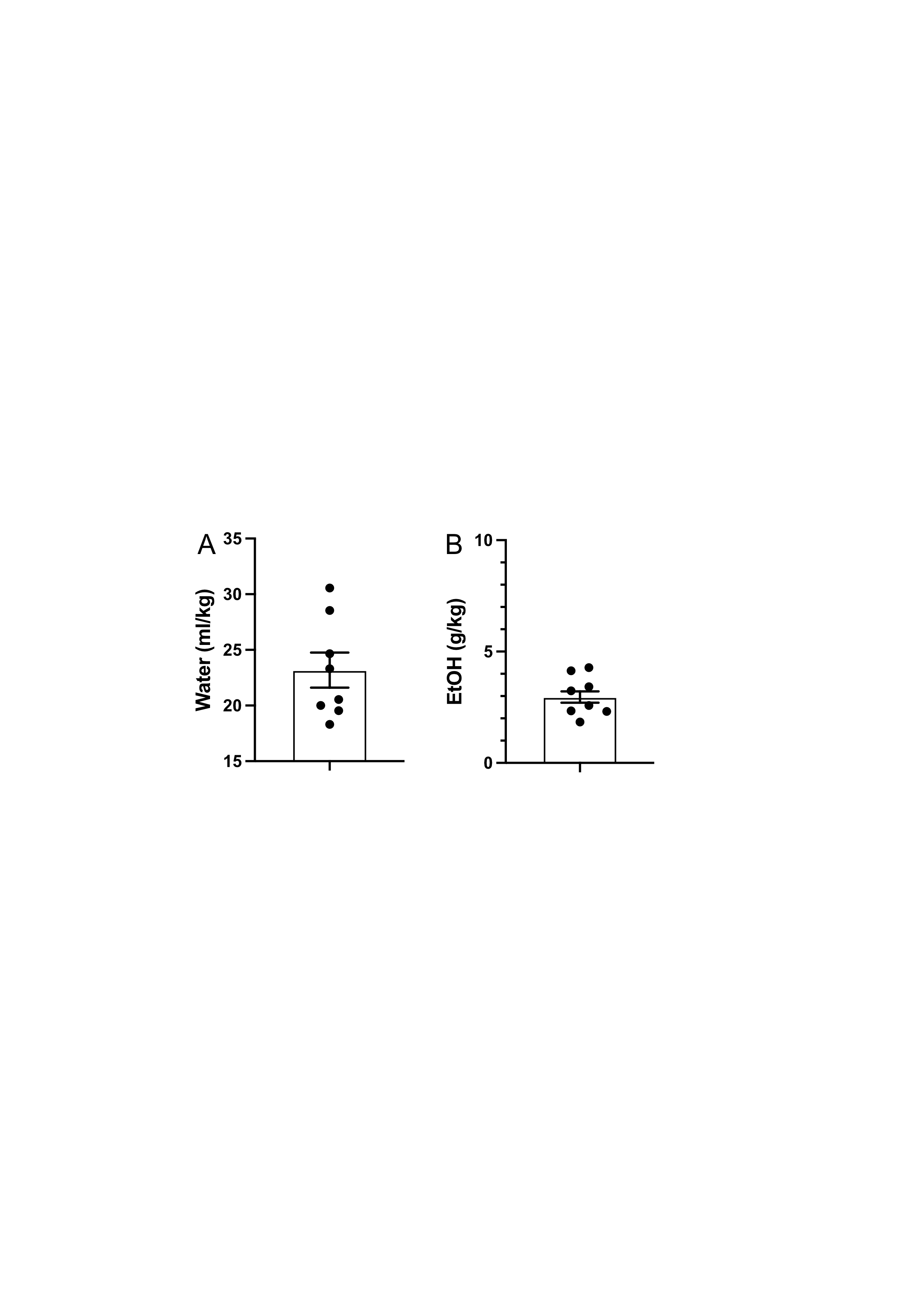
**

Supplement: Supplementary Figure 1 — D1-MSNs sEPSC and evoked EPSP measurements after ChI optogenetic stimulation. (A) Average sEPSCs amplitudes before (Pre, white circles) and after (Post, blue circles) ChI optogenetic stimulation in D1-MSNs ChAT.ChR2 mice (n = 14). (B) Average sEPSCs amplitudes before (Pre, white circles) and after (Post, gray circles) light stimulation of ChIs in D1-MSNs TdTomato mice (n = 10). (C) Electrically-evoked EPSPs in ChAT-ChR2 D1-MSNs before (Pre, white circles), during (white circles, blue bar), and after (Post, blue circles) ChI optogenetic stimulation (n = 10). (D) Electrically-evoked EPSPs in TdTomato control D1-MSNs before (Pre, white circles), during (white circles, blue bar), and after (Post, gray circles) ChI optogenetic stimulation (n = 7). *p < 0.05. [file Data_Sheet_1.docx]
